# Defluorination of HFCs by a magnesium reagent†

**DOI:** 10.1039/d4dt00636d

**Published:** 2024-03-28

**Authors:** Daniel J. Sheldon, Joseph M. Parr, Mark R. Crimmin

**Affiliations:** a Department of Chemistry, Molecular Sciences Research Hub, Imperial College London London W12 0BZ UK m.crimmin@imperial.ac.uk

## Abstract

Reaction of a series of HFCs with a main group reagent containing a Mg–Mg bond results in defluorination to form the corresponding magnesium fluoride complex. In the case of 1,1,1,2-tetrafluoroethane (HFC-134a) generation of the fluoride occurs alongside selective formation of 1,1-difluoroethene. DFT calculations have been carried out to better understand the selectivity and compare the barriers for sp^3^ C–F bond activation with sp^3^ C–H bond activation in this system.

## Abstract 2

Reaction of a series of HFCs with a main group reagent containing a Mg–Mg bond results in defluorination to form the corresponding magnesium fluoride complex.

## Graphical abstract

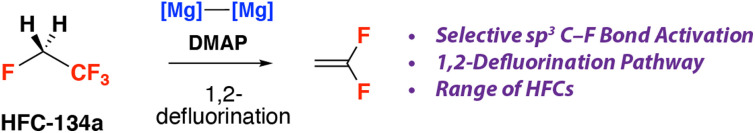

Due to their unique chemical and physical properties hydrofluorocarbons (HFCs) have been widely used as 3^rd^ generation refrigerants. The largest use HFC for this purpose is 1,1,1,2-tetrafluoroethane (HFC-134a). Perhaps as a result, HFC-134a has become one of the most abundant fluorinated gases in the atmosphere. HFC-134a has a 100-year global warming potential (GWP_100_) of 1530 and is contributing to climate change. Legislation is now in place to limit the use of HFCs globally.^1–5^ The planned phase-down of HFCs is however a gradual process; developed countries have committed to phase-down by 85% by 2036, with other countries committing to 80% by the late 2040s.^6^ Consequently, HFCs may continue to be emitted for a long time. There is a need to develop processes to repurpose HFCs, ideally with destruction and removal of the fluorine content of these molecules.

Reactions of 1,1,1,2-tetrafluoroethane nearly always involve pathways that result in the formal elimination of an equivalent of HF (Fig. 1). Strong bases, organometallic reagents, or heterogeneous catalysts react with HFC-134a to form 1,1,2-trifluoroethene.^7–9^ This chemoselectivity is driven by the strong sp^3^ C–F bond strengths of both fluorinated sites, and the relative acidity of the adjacent sp^3^ C–H bonds. In the case of reactions with organometallic compounds, *in situ* deprotonation of 1,1,2-trifluoroethane to form a trifluorovinyl moiety is common. For example, it has been reported that the reaction of HFC-134a with 2 equiv. *n*-BuLi at −78 °C forms trifluorovinyllithium. This species, which decomposes if warmed to room temperature, can react at −78 °C with a wide range of electrophiles including metal halides, main group halides, CO_2_, aldehydes and epoxides.^7,8,10–26^ Other reports include the transfer of the trifluorovinyl group onto zinc chlorides for application in palladium-catalysed Negishi cross-coupling reactions.^19,27–31^

**Fig. 1:**
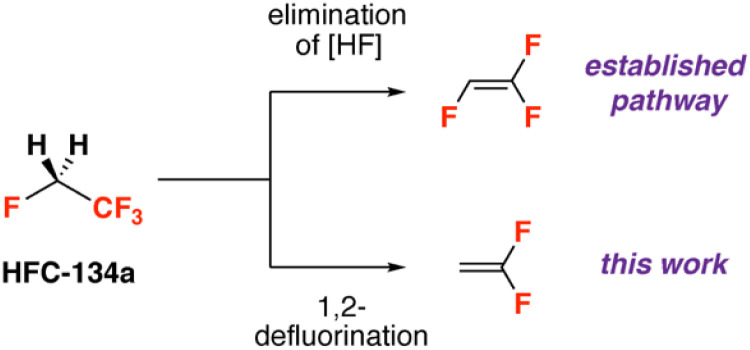
Known HF elimination pathway for HFC-134a and this work.

To the best of our knowledge there are no examples of reactions of HFC-134a which involve solely sp^3^ C–F bond activation. In this communication, we show that reaction of HFC-134a with a main group reagent containing a Mg–Mg bond leads to exclusive formation of 1,1-difluoroethene due to the formal 1,2-elimination of two F atoms from the substrate. This is a rare example of selective sp^3^ C–F bond activation of this industrially important HFC.

HFC-134a (1 bar, 25 °C, approx. 7 equiv.) was added to a degassed C_6_D_6_ solution of 1 and 4-dimethylaminopyridine (DMAP, 2 equiv.). The reaction mixture was agitated and left to proceed for 16 h at 25 °C, during which time the dark red solution turned yellow. ^1^H and ^19^F NMR spectroscopy of the resultant reaction mixture reveal the formation of 2 (86% yield) and 1,1-difluoroethene (Scheme 1). 2 has been previously reported and characterised by our group, and shows a diagnostic resonance at *δ* = −183.9 ppm in the ^19^F NMR spectrum.^32^ 1,1-Difluoroethene has a resonance at *δ* = −81.8 ppm, in accordance with data in the literature.^33^ Due to its volatility and partitioning between solution and reaction headspace, the amount of 1,1-difluoroethene was not quantified, subsequent onwards reaction however, support generation in reasonable yields (*vide infra*).

**Scheme 1:**
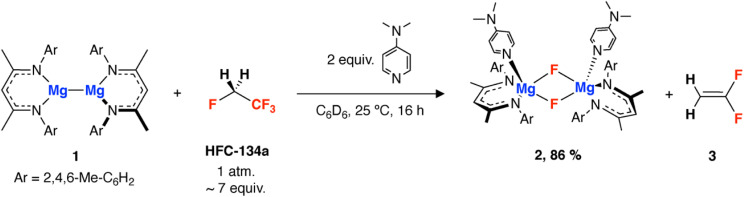
Defluorination of HFC-134a by a magnesium reagent (1) and DMAP.

Jones and co-workers have shown that the magnesium nucleophile used in this reaction, 1,^34,35^ displays enhanced reactivity on the addition of a Lewis base,^36,37^ while we have recently demonstrated its use in the defluorination of PTFE.^32^ Defluorination of HFC-134a with 1 can also occur in the absence of DMAP, but requires heating to 80 °C, and results in 30% yield of an analogue of 2 which does not have DMAP coordinated.

To gain further insight into this unique reactivity, the mechanism of defluorination of HFC-134a was studied using computational techniques (DFT). Binding of DMAP to 1 was assumed to be fast and reversible under the reaction conditions, reinforced by observed fluxionality in ^1^H NMR spectroscopic data for 1·DMAP suggesting that the DMAP can move rapidly between Mg centres.^32,36,37^

The reaction is initiated by attack of 1·DMAP at the sp^3^ C–F bond of the –CH_2_F group of HFC-134a, *via*TS-1 (Δ*G*^‡^_298 K_ = 19.5 kcal mol^−1^), to form Int-1. This reactivity mode for 1·DMAP has been established previously in the defluorination of poly(tetrafluoroethene) (PTFE).^32^TS-1 is asymmetric, the three-coordinate Mg centre of 1·DMAP inserts into the C–F bond, with the fluoride ion migrating into a bridging position between both Mg sites. One way to conceptualise this reaction is as a bimetallic oxidative addition of the C–F bond of the HFC to 1·DMAP. Calculated *NPA* charges in 1·DMAP reveal the 3-coordinate Mg atom has a less positive charge (+0.84) compared to the four-coordinate Mg atom (+1.07), and hence attack originates from the three-coordinate Mg atom. It has been proposed that polarisation and stretching of the Mg–Mg bond in 1·DMAP leads to enhanced reactivity compared to the symmetric species 1 or 1·DMAP_2_.^32,37^ NBO calculations reveal a flow of charge from 1·DMAP to the fluorine atom of HFC-134a as TS-1 is traversed (see ESI†). Int-1 undergoes fluoride elimination *via*TS-2 (Δ*G*^‡^_298 K_ = 6.6 kcal mol^−1^) to form 1,1-difluoroethene, and reaction of the magnesium fluoride intermediate with a second equivalent of DMAP forms 2. This second step effectively breaks one of the C–F bonds of the CF_3_ group of HFC-134a completing the formal 1,2-defluorination (Fig. 2).

**Fig. 2:**
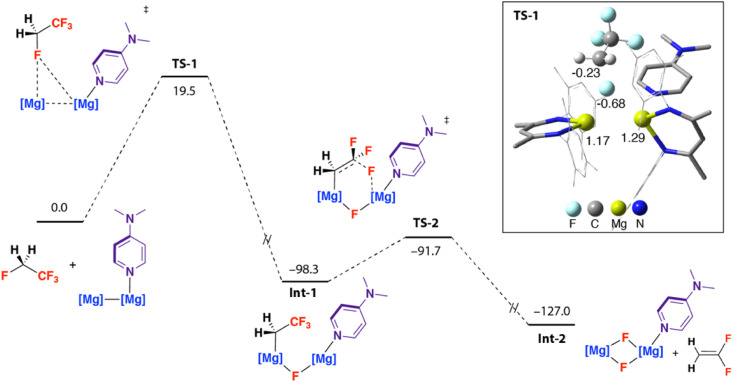
Calculated reaction pathway for defluorination of HFC-134a by 1 + DMAP. Gibbs energies in kcal mol^−1^. G09:B3PW91-GD3BJ/6-311+G*(C,H,N,F)/SDDAll(Mg)//B3PW91-GD3BJ/6-31G**(C,H)/6-311+G*(N,F)/SDDAll(Mg). Inset, representation of TS-1 annotated with *NPA* charges on key atomic sites.

The mechanism proposed based on DFT calculations is perhaps intuitive. The fluorophilicity of 1 likely results in selective sp^3^ C–F bond activation over sp^3^ C–H bond activation, while the trends in bonds strengths would favour reaction at the isolated sp^3^ C–F bond.^38^ Alternative mechanisms were calculated and found to lead to transition states that were less energetically accessible than TS-1. Deprotonation of the sp^3^ C–H bond of HFC-134a with 1·DMAP was calculated to occur *via*TS-3 with Δ*G*^‡^_298 K_ = 27.1 kcal mol^−1^. Attack at a fluorine atom of the CF_3_ group of HFC-134a was calculated to occur *via*TS-4, Δ*G*^‡^_298 K_ = 26.5 kcal mol^−1^. A concerted pathway involving simultaneous breaking of two sp^3^ C–F bonds was also considered but a suitable transition state could be not be located.

Consideration of selectivity in the possible bond breaking events for HFC-134a raises the question as to how small changes to structure might affect the reaction outcome. A range of hydrofluoroethanes including 1,1,1-trifluoroethane (HFC-143a, GWP_100_ = 5810), 1,1-difluoroethane (HFC-143a, GWP_100_ = 164) and 1,1,1,2,2-pentafluoroethane (HFC-125, GWP_100_ = 3740) are widely available and used in the refrigeration sector.^9,39^ Along with HFC-134a, this series of substrates represents a group with systematic changes in number and position of fluorine atoms.

The computational model was extended to examine the activation barriers for sp^3^ C–F activation and sp^3^ C–H activation of this range of HFCs. Several trends are consistent across the series: (i) in general activation barriers increase with lower fluorine content of the substrate, (ii) in cases where multiple C–F bonds are present there is preference for CFH_2_ > CF_3_ and CF_2_H > CF_3_ groups – reflecting the known trends in sp^3^ C–F bond strengths,^38^ and (iii) sp^3^ C–F activation is consistently a more facile process than sp^3^ C–H bond activation (Fig. 3). For example, the activation barriers for sp^3^ C–F bond activation (TS-1) increase in the order 1,1,1,2,2-pentafluoroethane (HFC-125, Δ*G*^‡^_298 K_ = 20.5, 25.8 kcal mol^−1^), 1,1,1-trifluoroethane (HFC-143a, Δ*G*^‡^_298 K_ = 29.6 kcal mol^−1^), 1,1-difluoroethane (HFC-152a, Δ*G*^‡^_298 K_ = 32.7 kcal mol^−1^). For 1,1,1,2,2-pentafluoroethane, there are two possible isomeric transition states for C–F bond activation, with attack of 1·DMAP occurring at either the CF_2_H or CF_3_ bond of the substrate, the lowest energy pathway involves defluorination of the difluoromethyl site.

**Fig. 3:**
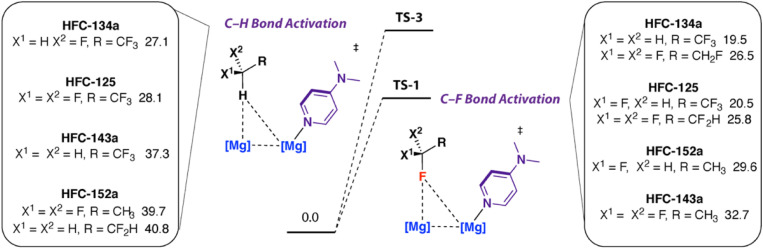
Calculated barriers for C–F and C–H activation of HFCs with 1 + DMAP. Gibbs energies in kcal mol^−1^. G09:B3PW91-GD3BJ/6-311+G*(CH,N,F)/SDDAll(Mg)//B3PW91-GD3BJ/6-31G**(C,H)/6-311+G*(N,F)/SDDAll(Mg).

The calculations suggest that these HFCs should also undergo chemoselective defluorination under accessible conditions with 1 + DMAP. Reaction of 1,1,1,2,2-pentafluoroethane with 1 + DMAP at 25 °C in C_6_D_6_ for 5 days led to the formation of 2 in a 59% yield. Reaction of 1 + DMAP with 1,1-difluoroethane in C_6_D_6_ occurred slowly at 25 °C, but was complete within 16 h at 60 °C forming 2 in 41% yield. 1 + DMAP also reacts with 1,1,1-trifluoroethane in C_6_D_6_ to form 2 after 16 h at 60 °C (Scheme 2). The elevated temperatures required for these reactions are consistent with the trends in calculated activation energies. In all these cases, magnesium hydride side-products derived from sp^3^ C–H activation are not observed, suggesting selective reaction at the sp^3^ C–F bond.

**Scheme 2:**
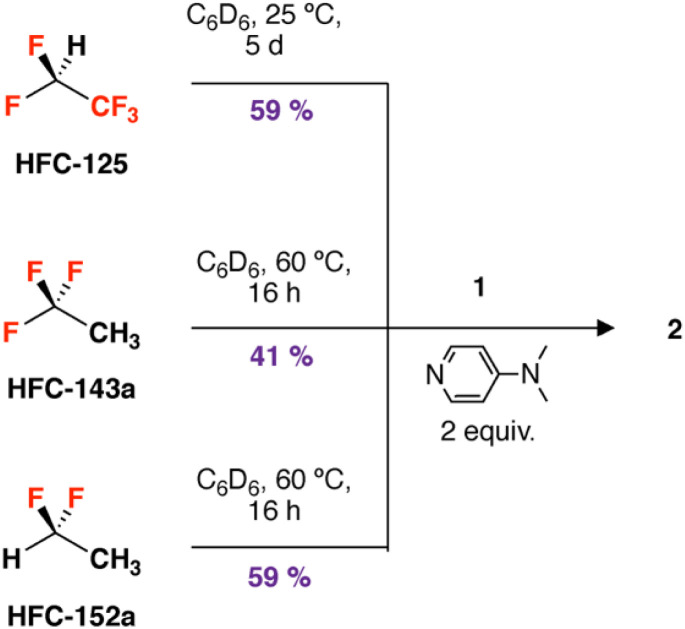
Defluorination of HFC-125, HFC-143a, HFC-152a by 1 + DMAP.

A minor fluoroalkene containing product could be identified from the reaction of 1,1,1,2,2-pentafluoroethane with 1 and DMAP, identified as α,β,β-trifluorostyrene-d_5_, formed in a 5–10% yield (based on 1 equivalent of 1). α,β,β-Trifluorostyrene-d_5_ likely derives from a defluorinative coupling of the HFC and the benzene-d_6_ reaction solvent. While the pathway for its formation remains unclear, generation of a fluoroethene intermediate cannot be ruled out at this stage.

In the case of both 1,1-difluoroethane and 1,1,1-trifluoroethane, we could not identify the organic products of these reactions. No obvious fluorine containing species were present following vacuum transfer of the volatiles as evidenced by ^19^F NMR spectroscopy. No products beyond 2 could be isolated from the residue and as such, the precise fate of the organic fragment in these reactions remains an open question. Nevertheless, the lack of clear formation of ethene products implies is that, without fluorine substitution at both carbon atoms of the ethane, the 1,2-elimination pathway may be switched off. In these cases, sp^3^ C–F bond activation would generate intermediate organomagnesium complexes likely to decompose through α-elimination pathways forming 2 along with unstable carbene fragments.

In terms of further use of the products from the defluorination of HFCs, we have previously shown that 2 can act as a nucleophilic source of fluoride, capably of fluorinating a small array of highly activated electrophiles.^32^ In the case of HFC-134a, the volatile product 1,1-difluoroethene could be separated from 2 by vacuum transfer, allowing further derivatisation by nucleophilic substitution (Scheme 3). 1,1-Difluoroethene was defluorosilylated upon reaction with the lithium silanide 4 to form the organosilicon compound 5 in 40% (NMR yield, over two steps based on 1 as the limiting reagent). 5, a known compound, was characterised by a diagnostic resonance in the ^19^F NMR spectrum at *δ* = −103.0 ppm.^40^ Related defluorosilylation reactions of fluoroalkenes including difluoroethene have been previously reported.^41–43^

**Scheme 3:**
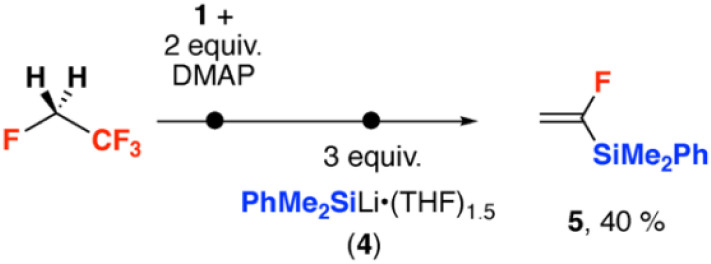
Two-step derivatisation of HFC-134a *via* 1,2-defluorination and nucleophilic substitution. Yield determined by ^19^F NMR spectroscopy.

In summary, we report the defluorination of a range of HFC refrigerants using a magnesium reagent. In the case of 1,1,1,2-tetrafluoroethene (HFC-134a), the largest commercial HFC, a highly unusual pathway for 1,2-defluorination to form 1,1-difluoroethene was observed. This pathway complements established reaction patterns which nearly always result in formation of 1,1,2-trifluoroethene through elimination of an equivalent of HF. DFT calculations have been used to compare the chemoselectivity in these systems. sp^3^ C–F bond activation universally occurs with lower barriers than sp^3^ C–H bond activation, likely due to participation of fluorophilic magnesium sites in the key transition state for bond breaking. The development of mechanistic understanding in reactions that defluorinate HFCs has the potential to underpin future approaches to repurpose these potent greenhouse gases.

## Conflicts of interest

There are no conflicts to declare.

## Supplementary Material

DT-053-D4DT00636D-s001

DT-053-D4DT00636D-s002
