# Supplementary material for: Defluorination of HFCs by a magnesium reagent
Source: Dalton Trans. 2024 Mar 28;53(15):6524–8. doi: 10.1039/d4dt00636d (PMC11019403; doi:10.1039/d4dt00636d)
Supplement: DT-053-D4DT00636D-s002 [file DT-053-D4DT00636D-s002.pdf]

Electronic Supplementary Material (ESI) for Dalton Transactions.
This journal is © The Royal Society of Chemistry 2024

Supporting information for:

Defluorination of HFCs by a Magnesium Reagent

Daniel J. Sheldon,[a] Joseph M. Parr,[a] and Mark R. Crimmin*[a]

*Corresponding author. Email: m.crimmin@imperial.ac.uk

[a] Molecular Sciences Research Hub, Department of Chemistry, Imperial College London, 82 Wood Lane, White City, Shepherds Bush, London, W12 0BZ, UK.

Contents

1. General Experimental……………………………………………………………1
2. Defluorination Procedures…………………………………………………………2
   2.1. Defluorination of HFC-134a with 1 and 4-(dimethylamino)pyridine..2
   2.2. Defluorination of HFC-134a with 1……………………………………4
   2.3. Defluorination of HFC-143a with 1 and 4-(dimethylamino)pyridine..5
   2.4. Defluorination of HFC-152a with 1 and 4-(dimethylamino)pyridine..5
   2.5. Defluorination of HFC-125 with 1 and 4-(dimethylamino)pyridine….6
3. Trapping Reactions of 3……………………………………………………………..8
4. Computational Methods……………………………………………………………..9
   4.1. Discussion of Computational Model……………………………………10
   4.2. Alternative Mechanisms……………………………………………….……11
   4.3. NBO Data for TS-1……………………………………………………………13
   4.4. Images of Transition States…………………………………………...……14
   4.5. Assessment of Functional…………………………………………....………15
   4.6. Assessment of Basis Set………………………………………………….…..16
   4.7. DFT Calculated NMR Spectra of 3……………………………...……16
   4.8. Cartesian Coordinates………………………………………………………...17
5. ^{19}F NMR Spectra……………………………………………………………………53
6. References…………………………………………………………….……………55

1. General Experimental

Standard Schlenk line and glovebox techniques were used for all manipulations under an inert atmosphere of dinitrogen or argon unless otherwise stated. NMR scale reactions were performed in J. Young NMR tubes equipped with internal standard capillaries of ferrocene (^{1}H NMR spectroscopy) and prepared in a glovebox. An MBraun Labmaster glovebox was used, operating at <0.1 ppm H_2O and <0.1 ppm O_2. ^{1}H, ^{13}C, and ^{19}F NMR spectra were recorded on Bruker 400 MHz or 500 MHz machines, and referenced against $SiMe_4$ (^{1}H, ^{13}C), $CFCl_3$ (^{19}F), H_3PO_4 (^{31}P). NMR data were processed using the MestReNova software package. Solvents were dried over activated alumina from a solvent purification system (SPS) based upon the Grubbs design and de-gassed before use. Glassware was dried for 12 hours prior to use at 120 °C. Benzene-d_6 was de-gassed and stored over 3 Å molecular sieves before use. All reagents were acquired from Sigma Aldrich, Fluorochem or Alfa Aesar and used without further purification unless specified. **1** was prepared following the literature procedure.[1] HFC-134a, HFC-143a, HFC-152a and HFC-125 were donated by Apollo Scientific and used without any further purification.

2. Defluorination Procedures

2.1. Defluorination of HFC-134a with 1 and 4-(dimethylamino)pyridine (DMAP)

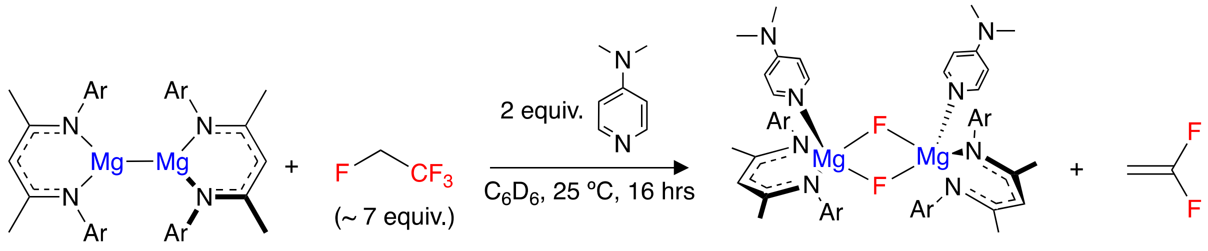

1 **2, 86 %** **3**

Ar = 2,4,6-Me-C_6H_2

Scheme S1: Defluorination of HFC-134a with **1** + DMAP.

In an N_2 filled glovebox, 10 mg (0.014 mmol) of **1** and 0.028 mmol of DMAP (140 µL of 0.2 M C_6D_6 stock solution, 2 equiv.) was dissolved in 0.6 mL of C_6D_6, added to a J. Young NMR tube equipped with a ferrocene capillary internal standard, and a t=0 ^{1}H NMR spectrum was recorded. The solution was degassed, and HFC-134a added (1 bar, 25 °C, 2.2 mL, approx. 7 equiv.). The J. Young tube was inverted multiple times and left overnight. After 16 hours, the solution had gone from red to pale yellow. ^{1}H and ^{19}F NMR spectra were recorded. The 86 % yield of the product **2** was determined *in situ* by integral comparison to the ferrocene internal standard in the ^{1}H NMR spectrum. This yield was cross-referenced by ^{19}F NMR spectroscopy, through addition of a fluorinated internal standard (1,2-difluorobenzene) at the end of the reaction, and comparison of the integral to that of **2**. **2** has been fully characterised by our group in a previous publication, and the multinuclear NMR data can be found below.[2] **3** was identified by a multiplet signal in the ^{19}F NMR spectrum at resonance at δ –81.8 ppm, matching the data in the literature.[3]

Characterisation of 2, in accordance with data in the literature.[2]

^{19}F NMR (C_6D_6, 100 MHz, 298 K): δ -183.9 (s, Mg–**F**).

^{1}H NMR (C_6D_6, 400 MHz, 298K): 1.77 (s, 12H, NCC**H**$_3$), 2.01 (s, 24H, ortho-C**H$_3$**), 2.20 (s, 12H, DMAP N(C**H$_3$**)$_2$), 2.33 (s, 12H, para-C**H$_3$**), 5.02 (s, 2H, CH_3C(C**H**)CCH_3), 6.04 (d, 4H, $^3J_{H-H}$ = 4.9 Hz, DMAP Ar-**H**), 6.95 (s, 8H, Ar-C**H**), 8.16 (d, 4H, DMAP Ar-**H**).

Characterisation of 3, in accordance with data in the literature.[3] **3** was characterised *in-situ* as a mixture with unreacted HFC-134a. All attempts to separate the gases by low-temperature distillations were unsuccessful. ^{1}H and ^{13}C NMR data for **3** matches that calculated by DFT (Section 4).

^{19}F NMR (C_6D_6, 100 MHz, 298 K): δ –81.8 ppm (m)
^{1}H NMR (C_6D_6, 400 MHz, 298 K): 3.30 (m, 2H, CF_2C**H**$_2$).
^{13}C NMR (C_6D_6, 125 MHz, 298 K): 63.0 (CF_2**C**H_2), 161.0 (**C**F_2CH_2).

Equivalents of DMAP:

In our previous work on the 1,2-defluorination of PTFE with **1** + DMAP, we established that the process required 2 equivalents of DMAP per equivalent of **1** in order to drive the reaction to the thermodynamic product of **2**, and the same methodology has been used here for the 1,2-defluorination of HFC-134a. We found that when just 1 equiv. of DMAP is used in this reaction, two signals are present in the ^{19}F NMR spectrum, [^{Mes}*BDI*Mg(F)]$_3$ at δ -203.5 ppm (^{Mes}*BDI* = (2,4,6-Me-C_6H_2NCMe)$_2$CH),[4] and a signal at δ -192.3 proposed to be the single DMAP coordinated product ^{Mes}*BDI*Mg(F)–Mg(F)(DMAP)*BDI*^{Mes}. Addition of a further equivalent of DMAP resolves this mixture to form only **2**.

2.2. Defluorination of HFC-134a with 1

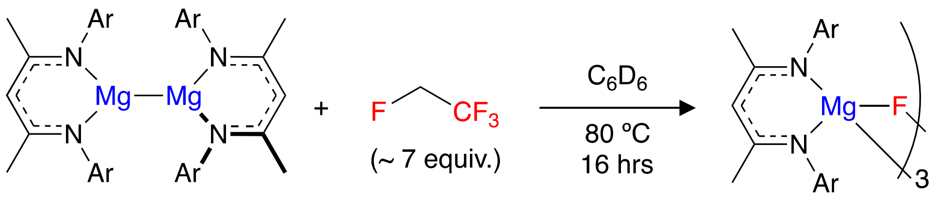

1

Ar = 2,4,6-Me-C_6H_2

30 %

Scheme S2: Defluorination of HFC-134a with **1**, with no DMAP.

In an N_2 filled glovebox, 10 mg (0.014 mmol) of **1** was dissolved in 0.6 mL of C_6D_6, added to a J. Young NMR tube equipped with a ferrocene capillary internal standard, and a t=0 ^{1}H NMR spectrum was recorded. The solution was degassed, and HFC-134a added (1 bar, 25 °C, 2.2 mL, approx. 7 equiv.). The J. Young tube was inverted multiple times and left to react at 80 °C. After 16 hours, the solution had gone from red to pale yellow. ^{1}H and ^{19}F NMR spectra were recorded. The 30 % yield of the magnesium fluoride was determined *in situ* by integral comparison to the ferrocene internal standard in the ^{1}H NMR spectrum. This yield was cross-referenced by ^{19}F NMR spectroscopy, through addition of a fluorinated internal standard (1,2-difluorobenzene) at the end of the reaction, and comparison of the integral to that of the magnesium fluoride. The magnesium fluoride product has been fully characterised by our group in a previous publication.[4]

2.3. Defluorination of HFC-143a with 1 and 4-(dimethylamino)pyridine (DMAP)

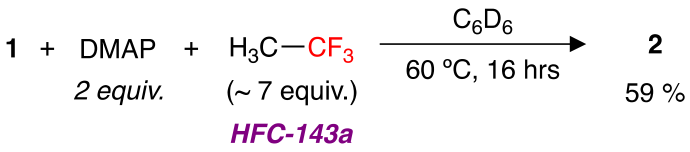

Scheme S3: Defluorination of HFC-143a with **1** + DMAP.

In an N_2 filled glovebox, 10 mg (0.014 mmol) of **1** and 0.028 mmol of DMAP (140 μL of 0.2 M C_6D_6 stock solution, 2 equiv.) was dissolved in 0.6 mL of C_6D_6, added to a J. Young NMR tube equipped with a ferrocene capillary internal standard, and a t=0 ^{1}H NMR spectrum was recorded. The solution was degassed, and HFC-143a added (1 bar, 25 °C, 2.2 mL, approx. 7 equiv.). The J. Young tube was inverted multiple times and left to react at 60 °C. After 16 hours, the solution had gone from red to pale yellow. ^{1}H and ^{19}F NMR spectra were recorded. The 59 % yield of the product **2** was determined *in situ* by integral comparison to the ferrocene internal standard in the ^{1}H NMR spectrum. The volatiles were distilled by vacuum transfer, analysis of the distillate by ^{19}F NMR spectroscopy revealed no clear fluorine-containing products.

2.4. Defluorination of HFC-152a with 1 and 4-(dimethylamino)pyridine (DMAP)

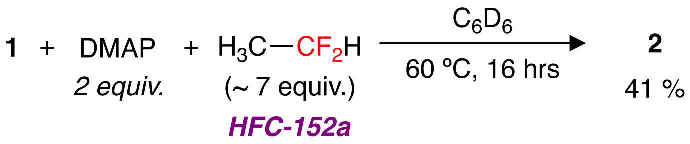

Scheme S4: Defluorination of HFC-152a with **1** + DMAP.

In an N_2 filled glovebox, 10 mg (0.014 mmol) of **1** and 0.028 mmol of DMAP (140 μL of 0.2 M C_6D_6 stock solution, 2 equiv.) was dissolved in 0.6 mL of C_6D_6, added to a J. Young NMR tube equipped with a ferrocene capillary internal standard, and a t=0 ^{1}H NMR spectrum was recorded. The solution was degassed, and HFC-152a added (1 bar, 25 °C, 2.2 mL, approx. 7 equiv.). The J. Young tube was inverted multiple times and left to react at 60 °C. After 16 hours, the solution had gone from red to pale yellow. ^{1}H and ^{19}F NMR spectra were recorded. The 41 % yield of the product **2** was determined *in situ* by integral comparison to the ferrocene internal standard in the ^{1}H NMR spectrum. The volatiles were distilled by vacuum transfer, analysis of the distillate by ^{19}F NMR spectroscopy revealed no clear fluorine-containing products.

2.5. Defluorination of HFC-125 with 1 and 4-(dimethylamino)pyridine (DMAP)

Room temperature reaction:

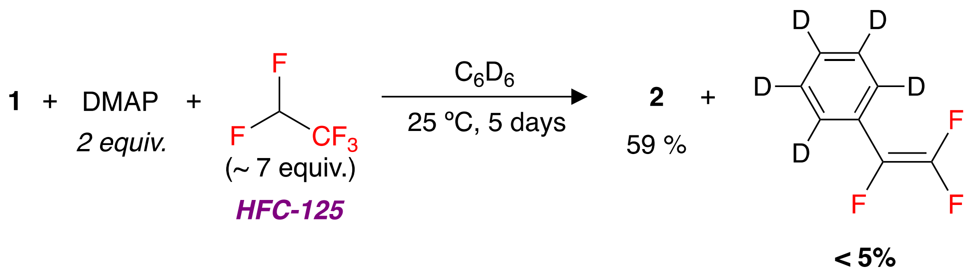

Scheme S5: Defluorination of HFC-125 with **1** + DMAP.

In an N_2 filled glovebox, 10 mg (0.014 mmol) of **1** and 0.028 mmol of DMAP (140 μL of 0.2 M C_6D_6 stock solution, 2 equiv.) was dissolved in 0.6 mL of C_6D_6, added to a J. Young NMR tube equipped with a ferrocene capillary internal standard, and a t=0 ^{1}H NMR spectrum was recorded. The solution was degassed, and HFC-125 added (1 bar, 25 °C, 2.2 mL, approx. 7 equiv.). The J. Young tube was inverted multiple times and left to react at 25 °C for 5 days. ^{1}H and ^{19}F NMR spectra were recorded. The 59 % yield of the product **2** was determined *in situ* by integral comparison to the ferrocene internal standard in the ^{1}H NMR spectrum. A trace amount (< 5 %, based on a 1:1 equivalence with **1**) of a second product was identified to be (α,β,β-trifluoro)styrene-d_5.

60 °C reaction:

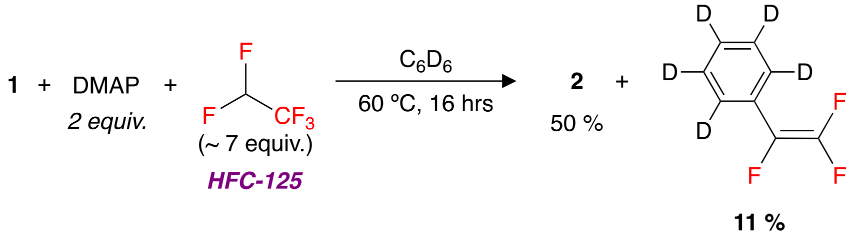

Scheme S6: Defluorination of HFC-125 with **1** + DMAP.

In an N_2 filled glovebox, 10 mg (0.014 mmol) of **1** and 0.028 mmol of DMAP (140 µL of 0.2 M C_6D_6 stock solution, 2 equiv.) was dissolved in 0.6 mL of C_6D_6, added to a J. Young NMR tube equipped with a ferrocene capillary internal standard, and a t=0 ^1H NMR spectrum was recorded. The solution was degassed, and HFC-125 added (1 bar, 25 °C, 2.2 mL, approx. 7 equiv.). The J. Young tube was inverted multiple times and left to react at 60 °C. After 16 hours, the solution had gone from red to pale orange. ^1H and ^{19}F NMR spectra were recorded. The 50 % yield of the product **2** was determined *in situ* by integral comparison to the ferrocene internal standard in the ^1H NMR spectrum. A second product was identified to be (α,β,β-trifluoro)styrene-d_5, in an 11 % yield, based on a 1:1 equivalence with **1**.

Characterisation of (α,β,β-trifluoro)styrene-d$_5$, in accordance with data in the literature for (α,β,β-trifluoro)styrene.[5]

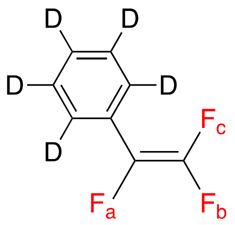

^{19}F NMR (C_6D_6, 100 MHz, 298 K): δ – 112.4 (dd, J_{FF} = 37.3, 82.5 Hz, F_b), δ -123.1 (dd, J_{FF} = 82.5, 111.0 Hz, F_c), δ -147.4 (dd, J_{FF} = 37.3, 111.0 Hz, F_a).

3. Trapping Reaction of 3

Reaction with $PhMe_2SiLi{\cdot}THF_{1.5}$ (4)

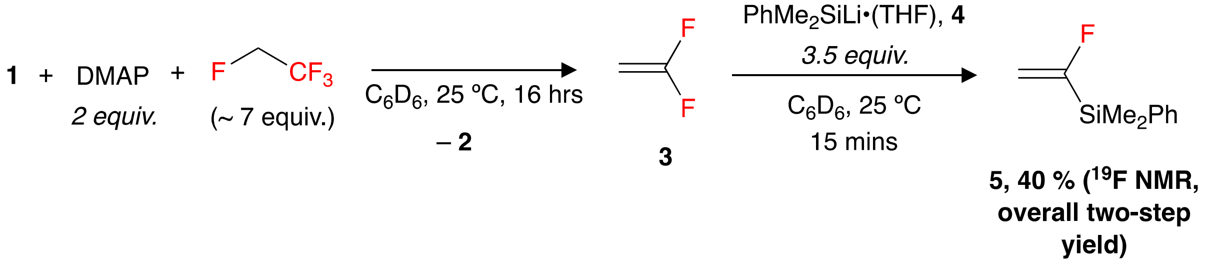

Scheme S7: Trapping reaction of 1,1-difluoroethene with $PhMe_2SiLi$.(THF).

In an N_2 filled glovebox, 10 mg (0.014 mmol) of **1** and 0.028 mmol of DMAP (140 μL of 0.2 M C_6D_6 stock solution, 2 equiv.) was dissolved in 0.6 mL of C_6D_6, added to a J. Young NMR tube equipped with a ferrocene capillary internal standard, and a t=0 ^{1}H NMR spectrum was recorded. The solution was degassed, and HFC-134a added (1 bar, 25 °C, 2.2 mL, approx. 7 equiv.). The J. Young tube was inverted multiple times and left overnight. After 16 hours, the solution had gone from red to pale yellow. ^{1}H and ^{19}F NMR spectra were recorded to confirm the completion of the reaction. The volatile species (*i.e.* 1,1-difluorethylene (**3**), excess unreacted HFC-134a and C_6D_6) were vacuum transferred into a new J Young NMR tube containing **4** (0.049 mmol, 10.5 mg, 3.5 equiv.). The J. Young tube was inverted multiple times, left for 15 minutes, and ^{1}H and ^{19}F NMR spectra were recorded. The ^{19}F NMR spectrum revealed consumption of the signal corresponding to **3** (δ – 81.8 ppm, m) and formation of a new double-doublet signal δ -103.0 ppm, which matched data in the literature for compound **5**.[6] The two-step 40 % yield of **5** was determined by addition of a fluorinated internal standard (1,2-difluorobenzene) at the end of the reaction, and comparison of the integral to that of **5**. Also visible in the ^{1}H NMR spectrum is $PhMe_2SiH$ (septet, δ = 4.63 ppm), from deprotonation of excess HFC-134a by **4**.

Characterisation of 5, in accordance with data in the literature.[6]

^{19}F NMR (C_6D_6, 100 MHz, 298 K): δ -103.0 (dd, $^3J_{FH}$ = 61.1 Hz, 32.5 Hz).

^{1}H NMR (C_6D_6, 400 MHz, 298K): δ 0.28 (s, Si<u>Me</u>$_2$Ph), 4.61 (dd, $^3J_{FH}$ = 61.1 Hz, $^2J_{HH}$ 2.9 Hz), δ 5.28 (dd, $^3J_{FH}$ = 32.5 Hz, $^2J_{HH}$ 2.9 Hz, C$\underline{H_2}$), δ 7.35-7.70 (m, Ar).

4. Computational Methods

DFT calculations were run using Gaussian 09 (Revision D.01)[9] using the using the B3PW91 density functional,[10-14] and an ultrafine integration grid (keyword int=ultrafine).[15] Geometry optimisations and frequency calculations were carried out using BS1, while single point energy calculations were then carried out at BS2 to obtain the final free energies.[16]

BS1 was built as follows. Mg centres were described with Stuttgart SDDAll RECPs and associated basis sets, while a hybrid basis set was used for the other atoms: 6-31g**(C, H)/6-311+g*(N, F).

BS2 was built as follows. Mg centres were described with Stuttgart SDDAll RECPs and associated basis sets, while 6-311+g* was used for all other atoms.

BS3 was built as follows. Mg centres were described with Stuttgart SDDAll RECPs and associated basis sets, while Ahlrichs triple-ξ basis set def2-TZVPP was used for all other atoms.[17]

Geometry optimisation calculations were performed without symmetry constraints. The Gaussian 09 default optimisation criteria were tightened to 10^{-9} on the density matrix and 10^{-7} on the energy matrix. The default numerical integration grid was also improved using a pruned grid with 99 radial shells and 590 angular points per shell. Frequency analyses for all stationary points were performed using the enhanced criteria to confirm the nature of the structures as either minima (no imaginary frequency) or transition states (only one imaginary frequency). Single point solvent corrections (benzene, $\varepsilon = 2.2706$) were applied using the polarizable continuum model (PCM) to free energies.[18] Single-point dispersion corrections using Grimme's D3 correction were applied to free energies, with Becke-Johnson damping applied for the B3PW91 functional.[19,20] Intrinsic reaction coordinate (IRC) calculations followed by full geometry optimisations on final points were used to connect transition states and minima located on the potential energy surface allowing a full energy profile (calculated at 298.15 K, 1 atm) of the reaction to be constructed.[21,22] The graphical user interface used to visualise the various properties of the intermediates and transition states was GaussView 5.0.9.[23] Natural Bond Orbital analysis was carried out using NBO 6.0.[24] DFT calculated NMR spectra of **3** were generated using keyword (nmr=giao) and referenced against TMS as standard in GaussView (ωB97XD/ cc-PVDZ $CDCl_3$ GIAO).

4.1. Discussion of Computational Model

The computational model used in this system is the same as that used in our previous work when studying C_2F_6.[2] DMAP binding to **1** is assumed to be fast and reversible. This assumption is reinforced by the ^{1}H NMR spectroscopic data at 25 °C of **1-(DMAP)** which reveals a broadened set of signals corresponding to 1 symmetrical ligand environment, demonstrating the fluxional behaviour of DMAP, where it is rapidly moving between Mg centres. Previous work by the Jones group came to the same conclusion.

In the reaction of **1** with HFC-134a, 2 equiv. of DMAP are used, as this drives the reaction to the thermodynamic product of **2.** However, the reaction of **1** and HFC-134a also proceeds at room temperature when only 1 equiv. of DMAP is added. This reaction formed a mixture of products, which was resolved to form the singular thermodynamic product of **2** by the addition of a second equiv. of DMAP.

Hence in the computational model, we consider **1-(DMAP)** as the active species and zero-energy point for the reaction with HFC-134a. The assumption is that DMAP can transfer to a different magnesium species with an insignificant energy penalty generating **1-(DMAP)** in situ. Calculations on a series of isodesmic reactions that suggest that DMAP can exchange between Mg centres in **1** and **2** with only a small energy penalty (Scheme S6). This model is more appropriate than considering the formal association and dissociation of DMAP to Mg atoms, as this method assumes DMAP can be free in solution, which is highly unlikely in practise, and occurs a significant and unrealistic energy penalty (~ 10 kcal mol^{-1}).

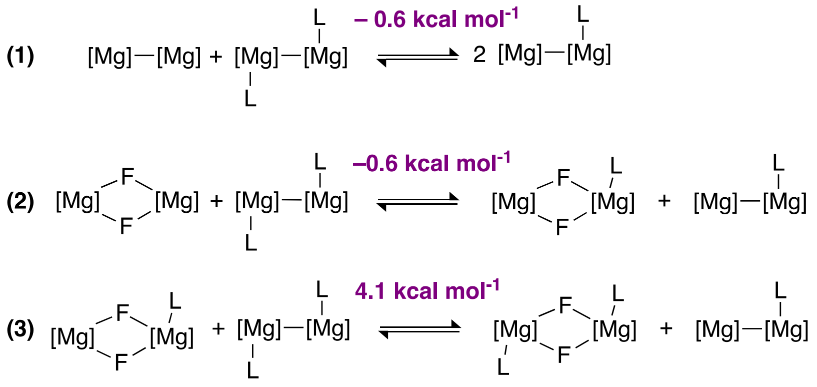

Scheme S8: Equilibria demonstrating the ease of DMAP transfer between different magnesium species present in the reaction mixture.

4.2. Alternative Mechanisms

We calculated transition states for alternative sites of nucleophilic attack by **1·DMAP**, such as at the proton of HFC-134a (**TS-3**, $\Delta G^{\ddagger}_{298\ K}$ = 27.1 kcal mol^{-1}), or at a fluorine atom of the CF_3 group (**TS-4**, $\Delta G^{\ddagger}_{298\ K}$ = 26.5 kcal mol^{-1}).

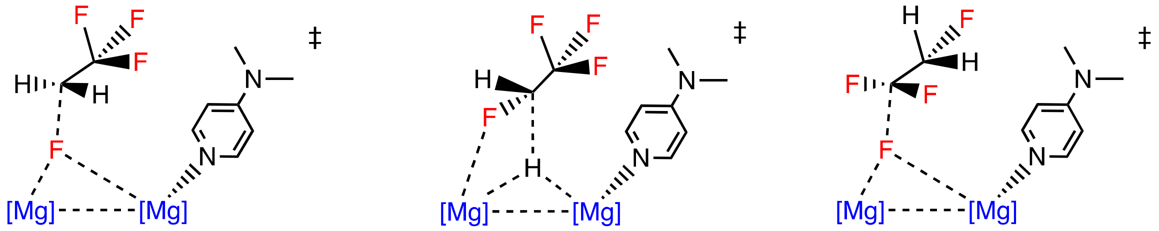

$\Delta G^{\ddagger}_{298K}$ = 19.5 kcal mol^{-1} **TS-1** | **$\Delta G^{\ddagger}_{298K}$ = 27.1 kcal mol^{-1}** **TS-3** | **$\Delta G^{\ddagger}_{298K}$ = 26.5 kcal mol^{-1}** **TS-4**

Figure S1: Alternative sites of nucleophilic attack.

A transition state where **1-(DMAP)** attacks the carbon atom of HFC-134a in an S_N2-type fashion could not be located, despite many attempts and variations of orientation.

We also explored the possibility of single electron transfer from the **1-(DMAP)** complex to HFC-134a as an alternative pathway for C–F activation. Calculations revealed that electron transfer to form a triplet radical-anion radical-cation pair was prohibitively high in energy compared to **TS-1** ($\Delta G^{\ddagger}_{298K}$ = 33.8 kcal mol^{-1}) (Figure S2).

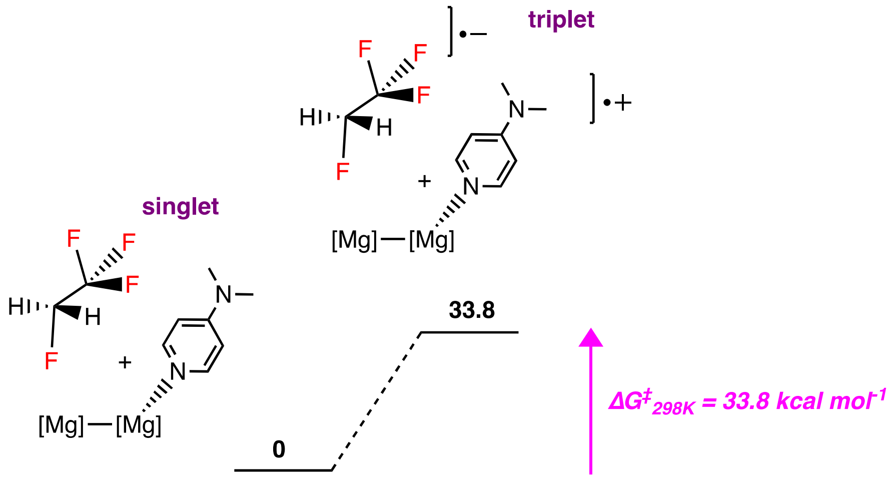

Figure S2: Calculated potential Energy Surface for single-electron transfer from **1-DMAP** to HFC-134a**.**

Efforts to find a concerted six membered-ring transition state wherein both C–F bonds break simultaneously were unsuccessful. Figure S3 shows some generic geometries used as inputs for our calculations. In all cases transition state optimisation calculations resulted in either ejection of HFC-134a or collapsed into stepwise transition states such as **TS-1** and **TS-2**. For **TS-concerted**, bong lengths a-d were systematically altered with α and β bond lengths fixed at 1.41 and 3.00 Å respectively (Table S1). The resulting geometries were subjected to transition state calculations; in no cases was a concerted transition state located.

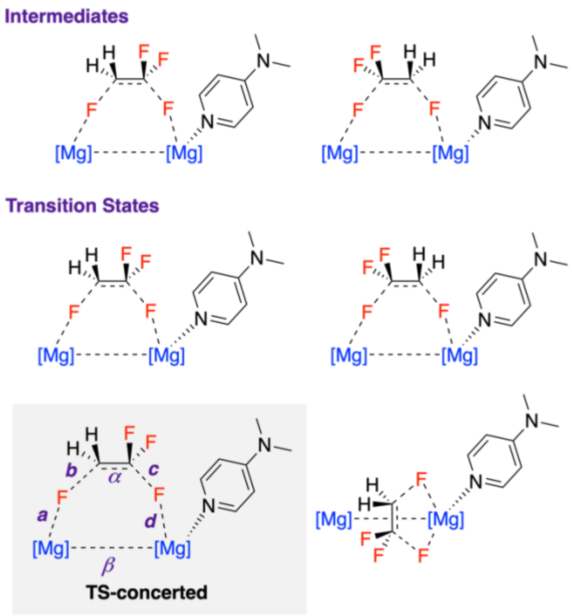

Figure S3: Proposed geometries to calculate six membered-ring intermediates and transition states.

Bond length (Å)							
**a**	**b**	**c**	**d**	**a**	**b**	**c**	**d**
1.90	1.70	1.70	1.90	2.05	1.60	1.60	2.05
1.95	1.70	1.70	1.95	2.05	1.65	1.65	2.05
2.00	1.70	1.70	2.00	2.05	1.70	1.70	2.05
2.05	1.70	1.70	2.05	2.05	1.75	1.75	2.05
2.10	1.70	1.70	2.10	2.05	1.80	1.80	2.05
2.15	1.70	1.70	2.15	2.05	1.70	1.60	2.05
2.00	1.70	1.70	1.90	2.05	1.70	1.65	2.05
2.00	1.70	1.70	1.95	2.05	1.70	1.75	2.05
2.00	1.70	1.70	2.00	2.05	1.70	1.80	2.05
2.00	1.70	1.70	2.05	2.05	1.65	1.60	2.05
2.00	1.70	1.70	2.10	2.05	1.65	1.65	2.05
2.00	1.70	1.70	2.15	2.05	1.65	1.75	2.05
2.05	1.60	1.60	2.05	2.05	1.65	1.80	2.05
2.05	1.65	1.65	2.05	2.05	1.65	1.85	2.05

Table S1: Bond lengths for attempted transition state optimisations of **TS-concerted**.

4.3. NBO Data for TS-1

NBO analysis was carried out and the relevant NPA charges for **TS-1** are tabulated below.

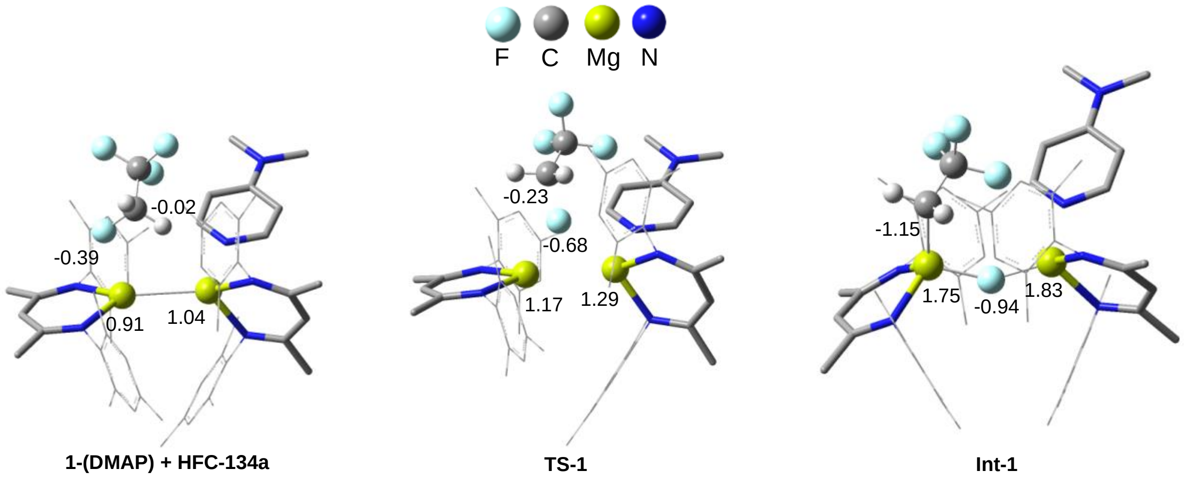

	1-DMAP + HFC-134a	TS-1	Int-1
**Mg**	0.91	1.17	1.75
**Mg_{DMAP}**	1.04	1.29	1.83
**F**	-0.39	-0.68	-0.94
**C**	-0.02	-0.23	-1.15
**N_{DMAP}**	-0.68	-0.69	-0.70

Table S2: NBO data for as **TS-1** is traversed.

4.4. Images of Transition States

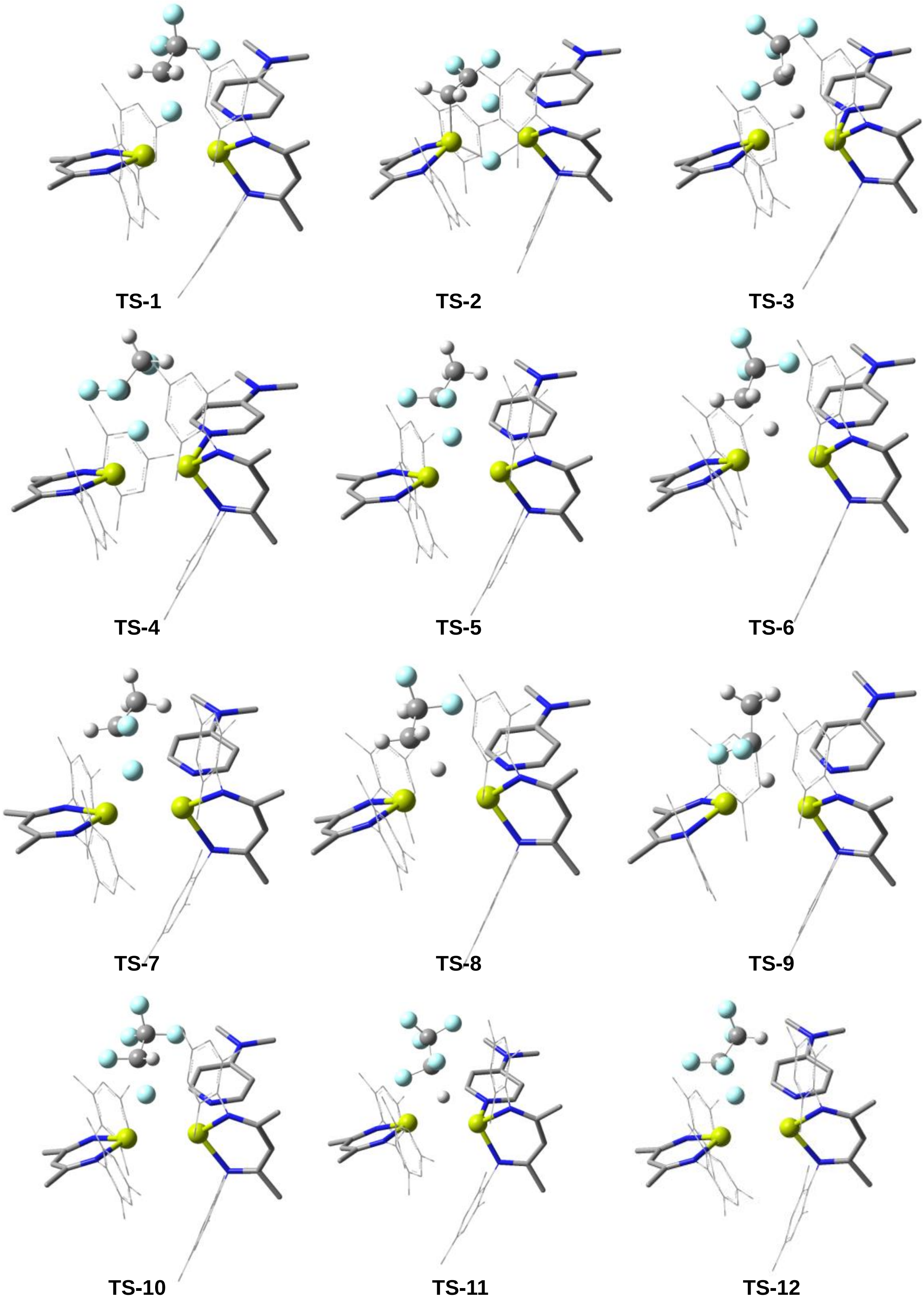

Figure S4: Depictions of TS-1 through to TS-12. Only important hydrogen atoms are displayed.

4.5. Assessment of the Functional

An assessment of the computational methodology was carried out by a series of functional benchmarking calculations (Table S3). The functionals tested were the B3PW91 density functional,[10–14] Minnesota hybrid-meta functional M06-2X,[15] the long-range corrected functional (with Grimme's D2 dispersion correction) ω-B97XD.[25,26] The same basis set and pseudopotential combination were maintained throughout. Single point solvent corrections (benzene, ε = 2.2706) were applied using the polarizable continuum model (PCM) to free energies.[18] Single point dispersion corrections using Grimme's D3 correction were applied to free energies in the cases of B3PW91 (also including Becke-Johnson damping), M06-2X and ω-B97X (noting that ω-B97X-D has Grimme's D2 dispersion correction built into the functional).[19] Consistent results were found across the different functionals with **TS-1** being found to have the lowest energy barrier for all methods. The chosen methodology, with B3PW91 as the functional, follows previous work in our group for C–F bond cleavage using Mg–Mg nucleophiles.[27]

It is noted that the functional benchmarking was carried out using the basis-set package BS2.

	$\Delta G^{‡}$ to **TS-1**	$\Delta G^{‡}$ to **TS-3**	$\Delta G^{‡}$ to **TS-4**
B3PW91	19.5	27.1	26.5
M062X	25.0	27.5	40.4
ωB97xD	27.7	32.8	38.5

Table S3: Relative free-energy barriers for the **TS-1**, **TS-3** and **TS-4** calculated for various density functionals. Free-energies in kcal mol^{-1}. BS2 was used for this functional assessment.

4.6. Assessment of Basis Sets

An assessment of the computational methodology was carried out by a series of basis set benchmarking calculations (Table S4). Calculated using B3PW91, with single point solvent (pcm, solvent=benzene) and dispersion (gd3bj) corrections.

	$\Delta G^{\ddagger}$ to **TS-1**	$\Delta G^{\ddagger}$ to **TS-3**	$\Delta G^{\ddagger}$ to **TS-4**
BS1	19.5	27.1	26.5
BS2	19.5	27.1	26.5
BS3	24.5	30.0	32.0

Table S4: Relative free-energy barriers for the **TS-1**, **TS-3** and **TS-4** calculated for various basis-sets. Free-energies in kcal mol^{-1}.

4.7. DFT calculated NMR spectra of 3

The NMR (^{1}H and ^{13}C) spectra of **3** was calculated using DFT (keyword nmr=giao) and referenced against TMS (ωB97XD/ cc-PVDZ $CDCl_3$ GIAO) in GaussView.

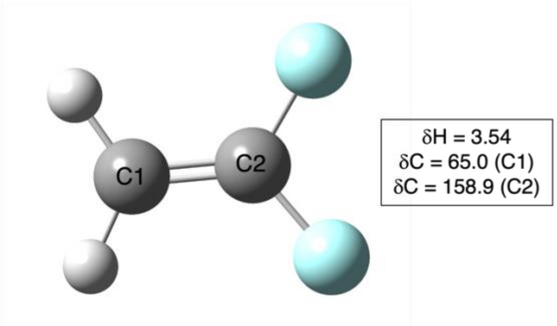

Figure S5: Annotated DFT calculated structure of **3** with calculated ^{1}H and ^{13}C NMR resonances in ppm.

4.8. XYZ Coordinates

1_DMAP.log

Mg	-0.002695	-1.228231	1.108162
N	1.141065	-2.531781	-0.070524
N	-1.475477	-2.691810	1.236300
C	1.936992	-4.680680	-0.938435
C	0.966616	-3.839966	-0.131443
C	-0.104366	-4.527916	0.482126
C	-1.294473	-3.980135	1.007898
C	-2.431369	-4.947189	1.272874
C	2.123278	-1.917307	-0.894967
C	3.427610	-1.683344	-0.432942
C	4.337328	-1.047968	-1.280681
C	3.983559	-0.610271	-2.553432
C	2.673755	-0.829097	-2.980292
C	1.738863	-1.476240	-2.175757
C	3.854338	-2.079145	0.957423
C	4.967477	0.127499	-3.425359
C	0.326741	-1.691986	-2.650981
C	-2.777202	-2.194177	1.520048
C	-3.153056	-1.909199	2.841353
C	-4.395086	-1.320692	3.078590
C	-5.269055	-1.005387	2.039981
C	-4.876198	-1.307692	0.737583
C	-3.640916	-1.887239	0.455265
C	-2.216000	-2.216785	3.980010
C	-6.583886	-0.318358	2.309899
C	-3.217663	-2.127328	-0.969626
H	2.969261	-4.494916	-0.627236
H	1.875729	-4.410997	-1.997924
H	1.721602	-5.745313	-0.838940
H	-0.091127	-5.606233	0.376343
H	-2.094636	-5.983228	1.212715

H	-3.229345	-4.800803	0.537482
H	-2.874397	-4.769307	2.257031
H	5.353334	-0.882672	-0.925679
H	2.370201	-0.488857	-3.968545
H	3.366287	-2.997248	1.292818
H	3.592801	-1.294109	1.676524
H	4.937773	-2.222708	1.003744
H	5.999669	-0.113758	-3.156071
H	4.827020	-0.116628	-4.482449
H	4.844560	1.212434	-3.322517
H	0.197598	-1.337379	-3.676743
H	0.036627	-2.746815	-2.608566
H	-0.379624	-1.147373	-2.013258
H	-4.684401	-1.099799	4.104287
H	-5.542948	-1.071969	-0.089071
H	-2.708651	-2.065460	4.944095
H	-1.851535	-3.248662	3.936772
H	-1.331143	-1.572362	3.942751
H	-6.471972	0.771518	2.264547
H	-6.969849	-0.566339	3.302885
H	-7.340695	-0.600609	1.572057
H	-4.075548	-2.069767	-1.643501
H	-2.730424	-3.096893	-1.104639
H	-2.502073	-1.358540	-1.284489
Mg	-0.325669	1.547245	0.493211
N	-1.407093	2.697900	-0.840094
N	0.877998	3.197716	0.891634
C	-2.219502	4.680756	-2.030823
C	-1.297329	4.003876	-1.036280
C	-0.341807	4.821828	-0.402973
C	0.721581	4.428769	0.429834
C	1.761958	5.481157	0.756414
C	-2.276740	1.940504	-1.673878
C	-3.584000	1.645522	-1.261050

C	-4.398648	0.891892	-2.105964
C	-3.937984	0.388055	-3.319774
C	-2.618901	0.655530	-3.685002
C	-1.778102	1.424020	-2.882703
C	-4.083673	2.111934	0.080334
C	-4.819189	-0.472907	-4.188605
C	-0.352188	1.694886	-3.288221
C	2.040883	2.846444	1.626118
C	2.066329	2.970159	3.023337
C	3.187900	2.513906	3.720281
C	4.258809	1.904062	3.070818
C	4.204210	1.782297	1.681751
C	3.119087	2.251635	0.945961
C	0.877462	3.535900	3.754557
C	5.432417	1.350209	3.838554
C	3.075559	2.095748	-0.550360
H	-3.265972	4.463615	-1.796893
H	-2.038927	4.298245	-3.040553
H	-2.074650	5.761686	-2.036810
H	-0.372842	5.872353	-0.665254
H	1.468279	6.462577	0.382165
H	2.719808	5.207718	0.300723
H	1.936011	5.550257	1.833756
H	-5.420771	0.682848	-1.796956
H	-2.233161	0.255907	-4.620968
H	-3.916378	3.183155	0.230365
H	-3.559195	1.582598	0.885832
H	-5.151254	1.905005	0.192365
H	-5.878257	-0.303008	-3.975501
H	-4.651018	-0.274168	-5.251236
H	-4.615141	-1.537089	-4.019856
H	-0.128759	1.240039	-4.256928
H	-0.143477	2.767763	-3.356481
H	0.352096	1.284820	-2.553868
H	3.210114	2.619441	4.803867
H	5.026403	1.302836	1.153735
H	0.632797	4.549669	3.422249
H	-0.013421	2.924787	3.567222
H	1.055097	3.563157	4.832699
H	5.450212	0.255023	3.779316
H	5.386283	1.630400	4.895611
H	6.383689	1.713594	3.437382
H	4.060191	1.834623	-0.943246
H	2.727863	3.007765	-1.044080
H	2.389646	1.287607	-0.837485
C	1.344689	-2.868923	3.345996
C	1.755648	-0.634243	3.595303
C	2.215196	-3.199972	4.364077
H	0.818434	-3.645893	2.796016
C	2.643661	-0.854283	4.629005
H	1.562348	0.376159	3.243550
H	2.352606	-4.244052	4.612752
H	3.128841	0.000466	5.080318
N	1.106406	-1.610335	2.950820
C	2.908204	-2.175536	5.048368
N	3.787773	-2.447274	6.054515
C	4.467873	-1.360653	6.732187
H	3.757077	-0.683535	7.221654
H	5.124464	-1.771462	7.498717
H	5.081931	-0.774214	6.038192
C	4.035394	-3.819459	6.450943
H	4.763570	-3.832410	7.261512
H	3.121165	-4.307661	6.810118
H	4.442358	-4.411020	5.621823

1.log

Mg	0.064736	-1.406745	0.023650

N	1.505150	-2.757906	-0.513068
N	-1.228436	-2.904636	0.541086
C	2.557870	-4.940012	-0.878779
C	1.388864	-4.077746	-0.449161
C	0.236147	-4.748366	0.000752
C	-0.978435	-4.204862	0.459153
C	-2.053541	-5.187429	0.875455
C	2.716037	-2.178854	-0.989475
C	3.733018	-1.843326	-0.084843
C	4.895472	-1.249092	-0.572575
C	5.054628	-0.937254	-1.919853
C	4.013146	-1.247619	-2.794503
C	2.844736	-1.864920	-2.352790
C	3.554330	-2.081453	1.391155
C	6.298683	-0.243294	-2.413469
C	1.726015	-2.184215	-3.310110
C	-2.491489	-2.458089	1.025230
C	-2.648661	-2.176252	2.392466
C	-3.872331	-1.684883	2.842930
C	-4.941570	-1.470509	1.973330
C	-4.755052	-1.749360	0.622208
C	-3.539390	-2.215963	0.125744
C	-1.502156	-2.393988	3.345448
C	-6.247294	-0.907861	2.474779
C	-3.341339	-2.412002	-1.354079
H	3.439424	-4.719922	-0.268666
H	2.834411	-4.724728	-1.915456
H	2.325052	-6.001510	-0.789785
H	0.291333	-5.829916	-0.006790
H	-1.712897	-6.218311	0.774860
H	-2.951892	-5.051806	0.265210
H	-2.352692	-5.013824	1.913643
H	5.690282	-1.004716	0.128349
H	4.113478	-1.004318	-3.850495
H	3.236509	-3.105313	1.611136
H	2.786348	-1.409399	1.794259
H	4.481773	-1.881346	1.933427
H	7.120523	-0.344039	-1.698965
H	6.632400	-0.650003	-3.372797
H	6.119511	0.828701	-2.560729
H	1.989282	-1.896521	-4.330958
H	1.479077	-3.251108	-3.307423
H	0.808400	-1.650731	-3.033122
H	-3.994292	-1.465968	3.901957
H	-5.572597	-1.579906	-0.074686
H	-1.151335	-3.431275	3.331356
H	-0.641824	-1.769987	3.073954
H	-1.790783	-2.144544	4.369496
H	-6.190162	0.182232	2.582901
H	-6.511991	-1.316725	3.454356
H	-7.066480	-1.126319	1.783847
H	-4.290915	-2.328541	-1.888546
H	-2.893647	-3.382397	-1.588967
H	-2.672261	-1.638890	-1.752734
Mg	-0.064737	1.406754	0.023302
N	-1.505219	2.757764	-0.513607
N	1.228484	2.904791	0.540200
C	-2.557989	4.939765	-0.879795
C	-1.388931	4.077621	-0.450073
C	-0.236165	4.748369	-0.000476
C	0.978473	4.204994	0.457932
C	2.053630	5.187679	0.873822
C	-2.716154	2.178574	-0.989725
C	-3.733041	1.843297	-0.084895
C	-4.895539	1.248913	-0.572340
C	-5.054828	0.936690	-1.919512
C	-4.013439	1.246815	-2.794357
C	-2.844990	1.864252	-2.352938

C	-3.554206	2.081841	1.391018
C	-6.298928	0.242580	-2.412806
C	-1.726365	2.183280	-3.310461
C	2.491597	2.458381	1.024311
C	2.648947	2.176941	2.391608
C	3.872676	1.685700	2.842055
C	4.941799	1.471069	1.972377
C	4.755104	1.749523	0.621198
C	3.539378	2.215986	0.124758
C	1.502568	2.394956	3.344680
C	6.247587	0.908560	2.473815
C	3.341131	2.411589	-1.355096
H	-3.439473	4.719837	-0.269524
H	-2.834645	4.724197	-1.916383
H	-2.325166	6.001288	-0.791117
H	-0.291355	5.829917	-0.008311
H	1.712973	6.218532	0.772981
H	2.951903	5.051885	0.263501
H	2.352914	5.014366	1.912021
H	-5.690274	1.004727	0.128735
H	-4.113876	1.003212	-3.850270
H	-3.236345	3.105758	1.610676
H	-2.786199	1.409886	1.794240
H	-4.481601	1.881906	1.933436
H	-7.120661	0.343419	-1.698191
H	-6.632817	0.649099	-3.372155
H	-6.119729	-0.829432	-2.559899
H	-1.989744	1.895322	-4.331205
H	-1.479411	3.250170	-3.308078
H	-0.808730	1.649854	-3.033429
H	3.994776	1.467095	3.901129
H	5.572556	1.579861	-0.075755
H	1.151769	3.432247	3.330358
H	0.642188	1.770902	3.073462
H	1.791323	2.145778	4.368756
H	6.190494	-0.181512	2.582172
H	6.512371	1.317637	3.453279
H	7.066699	1.126892	1.782756
H	4.290638	2.327992	-1.889664
H	2.893388	3.381907	-1.590209
H	2.672015	1.638347	-1.753438

2.log

Mg	1.998609	8.288993	5.883938
N	0.160965	8.462059	6.806864
C	-2.270404	8.312266	7.054194
H	-2.189495	7.735105	7.979600
H	-3.137817	7.964328	6.491785
H	-2.442915	9.353664	7.346624
Mg	4.245126	9.985403	7.143654
N	1.062223	8.334280	3.998050
C	-1.001256	8.215853	6.230684
N	2.238775	6.126754	5.831588
C	-1.159859	7.915917	4.860656
H	-2.173863	7.700363	4.545421
N	2.904502	2.023025	5.340629
C	-0.224273	8.098175	3.816483
N	6.132971	9.619759	6.396716
C	-0.779963	8.050968	2.405281
H	-0.184327	7.386858	1.772104
H	-0.735741	9.043982	1.946644
H	-1.817647	7.714552	2.398419
N	4.645517	12.054999	7.150281
C	0.204209	8.948195	8.139554
C	0.337313	8.061093	9.217393
C	0.438733	8.582383	10.508903
H	0.528835	7.892842	11.347128
C	0.459714	9.955222	10.747800

C	0.347699	10.816012	9.655421
H	0.368079	11.892510	9.815270
C	0.212040	10.337563	8.353212
C	0.420780	6.578521	8.966403
H	1.318726	6.347108	8.383125
C	0.101279	11.282645	7.187120
H	-0.695281	10.985568	6.498477
C	1.839035	8.766755	2.886442
C	1.853734	10.138632	2.570443
C	2.647628	10.579393	1.514581
H	2.661681	11.641576	1.277935
C	3.419771	9.700427	0.755512
C	3.383172	8.348385	1.083632
H	3.974175	7.643430	0.501043
C	2.617710	7.864178	2.146501
C	1.029429	11.108225	3.374755
H	-0.033716	10.844719	3.368792
C	2.640608	6.394651	2.478719
H	1.639029	6.006697	2.684094
C	1.271183	5.312304	5.390338
H	0.324270	5.789270	5.148028
C	1.430750	3.951226	5.226563
H	0.595570	3.368856	4.860185
C	2.678854	3.357392	5.520872
C	3.681447	4.220753	6.008895
H	4.670146	3.864471	6.265481
C	3.412906	5.567932	6.149198
H	4.165663	6.250943	6.527458
C	1.856144	1.176280	4.808005
H	0.972275	1.175458	5.456947
H	2.221912	0.151834	4.741322
H	1.548389	1.493712	3.803509
C	4.219482	1.473272	5.605217
H	4.989435	1.954083	4.988858

H	4.216263	0.408451	5.373104
H	4.498967	1.588513	6.659508
C	6.048758	14.064765	7.203501
H	5.624531	14.565988	6.327546
H	7.104608	14.329697	7.273975
H	5.521602	14.460470	8.076559
C	5.862759	12.563077	7.090392
C	7.038030	11.803812	6.886517
H	7.963772	12.366840	6.893629
C	7.142282	10.470247	6.436876
C	8.498126	10.034342	5.917400
H	8.803195	9.078776	6.353124
H	9.264176	10.782178	6.126413
H	8.447916	9.884468	4.833507
C	3.533669	12.941757	7.089312
C	3.063329	13.342895	5.824412
C	1.947482	14.172838	5.751525
H	1.582919	14.475360	4.771713
C	1.289541	14.626142	6.894672
C	1.777724	14.220899	8.133381
H	1.282820	14.567079	9.039391
C	2.880918	13.373089	8.253838
C	3.756066	12.867723	4.575099
H	3.694619	11.777863	4.510479
C	3.347816	12.939509	9.619270
H	4.436636	12.983340	9.711072
C	6.283176	8.368842	5.743320
C	6.657820	7.227811	6.467403
C	6.731901	6.002892	5.801276
H	7.035280	5.119176	6.360938
C	6.398287	5.877436	4.453956
C	6.008688	7.024613	3.762176
H	5.737025	6.951803	2.710651
C	5.951909	8.272602	4.380685

C	6.920420	7.327362	7.946950
H	6.005233	7.624028	8.470874
C	5.518414	9.496111	3.618591
H	4.501199	9.786313	3.904804
H	5.517973	9.305546	2.544455
H	6.163679	10.354400	3.827346
H	7.259234	6.369623	8.351372
H	7.675188	8.085606	8.179634
C	6.402215	4.536070	3.765814
H	5.375909	4.176976	3.618060
H	6.875506	4.590368	2.780604
H	6.937969	3.785821	4.356056
H	3.291507	13.298167	3.684070
H	4.818277	13.134956	4.569719
H	3.051577	11.904974	9.823969
H	2.908863	13.571921	10.396324
C	0.057163	15.488083	6.787642
H	-0.839064	14.872506	6.645452
H	-0.093665	16.084509	7.692076
H	0.120432	16.172487	5.936508
H	1.131053	12.124054	2.983858
H	1.360650	11.098978	4.416741
H	3.072778	5.819302	1.654916
H	3.246054	6.204712	3.371671
C	4.305203	10.208446	-0.354024
H	3.852719	11.061691	-0.867862
H	5.273342	10.540453	0.039519
H	4.501761	9.430218	-1.097126
H	-0.086649	12.301615	7.528190
H	1.033034	11.290973	6.610486
H	0.462292	6.022207	9.906819
H	-0.432869	6.211928	8.386979
C	0.651826	10.507568	12.137375
H	0.507854	9.733829	12.898216
H	1.666139	10.909401	12.254444
H	-0.049281	11.321114	12.347748
C	3.726458	9.135554	10.055193
C	3.812157	9.086151	11.432456
C	4.865576	9.754956	12.089544
C	5.794040	10.425432	11.261460
C	5.608296	10.424362	9.893880
N	4.590132	9.805036	9.282339
H	2.929181	8.623831	9.527326
H	3.049778	8.542757	11.974439
H	6.643956	10.954904	11.671618
H	6.300307	10.953628	9.242742
N	4.978752	9.756066	13.450078
C	6.063045	10.476396	14.086600
H	6.029309	11.548230	13.853811
H	5.982715	10.363365	15.167563
H	7.040689	10.085809	13.779519
C	3.975538	9.089753	14.256909
H	3.942821	8.013013	14.050495
H	4.217337	9.222555	15.311251
H	2.975583	9.505947	14.081992
F	3.359284	8.225535	7.316843
F	2.889733	10.029662	5.727662

dmap.log

C	-3.575389	1.216623	0.743949
C	-2.197213	1.362470	0.772310
C	-1.588964	2.330182	-0.052372
C	-2.461766	3.098996	-0.848352
C	-3.825343	2.857412	-0.787439
N	-4.404957	1.936689	-0.014225
H	-4.043260	0.467905	1.379461
H	-1.615792	0.728649	1.429763
H	-2.095485	3.875779	-1.507488

H	-4.497593	3.450143	-1.403950
N	-0.229483	2.512478	-0.078914
C	0.332552	3.604127	-0.846573
H	-0.021414	4.583818	-0.496804
H	1.418852	3.583250	-0.757071
H	0.086251	3.508394	-1.910145
C	0.609628	1.784706	0.850122
H	0.512186	0.702508	0.707508
H	1.652668	2.047509	0.672608
H	0.370679	2.018108	1.896965

int1.log

Mg	0.189453	-1.461174	0.013660
N	1.327641	-2.590844	-1.252439
N	-1.326169	-2.812589	0.043949
C	2.094839	-4.697727	-2.242613
C	1.110961	-3.891314	-1.418511
C	-0.006305	-4.588074	-0.918770
C	-1.199706	-4.057510	-0.373410
C	-2.404115	-4.973932	-0.350842
C	2.353210	-1.959353	-2.014144
C	3.644731	-1.778679	-1.497323
C	4.600802	-1.137201	-2.288498
C	4.305492	-0.651878	-3.559693
C	3.007998	-0.825167	-4.042143
C	2.027403	-1.468540	-3.292035
C	4.014192	-2.253295	-0.115459
C	5.339882	0.085032	-4.371848
C	0.631468	-1.643356	-3.830569
C	-2.603857	-2.290396	0.406576
C	-3.051382	-2.357225	1.734175
C	-4.243341	-1.717970	2.068032
C	-4.990138	-1.010756	1.125397
C	-4.529540	-0.973506	-0.188607
C	-3.340941	-1.598731	-0.564562
C	-2.236333	-3.071526	2.779744
C	-6.223661	-0.248136	1.535456
C	-2.832030	-1.491917	-1.976750
H	3.117055	-4.555637	-1.879949
H	2.082344	-4.360824	-3.284210
H	1.854317	-5.760992	-2.219770
H	-0.031882	-5.646723	-1.146643
H	-2.124764	-6.005093	-0.570178
H	-3.123037	-4.641766	-1.107778
H	-2.921355	-4.937302	0.610612
H	5.607461	-1.013056	-1.893294
H	2.751633	-0.447778	-5.029915
H	3.532871	-3.202682	0.132892
H	3.699302	-1.526280	0.641724
H	5.096707	-2.378882	-0.026158
H	5.244880	1.168175	-4.232841
H	6.354515	-0.197078	-4.077531
H	5.227329	-0.118198	-5.440675
H	0.545009	-1.224008	-4.835809
H	0.340043	-2.698233	-3.872582
H	-0.102126	-1.143348	-3.190215
H	-4.579398	-1.743457	3.101772
H	-5.090039	-0.424359	-0.941979
H	-2.689504	-2.959808	3.767246
H	-2.142433	-4.142292	2.565368
H	-1.224117	-2.660225	2.824575
H	-5.945296	0.711600	1.985864
H	-6.810419	-0.799281	2.276235
H	-6.867235	-0.038273	0.676476
H	-3.607374	-1.111558	-2.642826
H	-2.472573	-2.451327	-2.361494
H	-1.994221	-0.786649	-2.029708
Mg	-0.996154	1.988226	-0.041597

N	-1.787693	2.927701	-1.701067	H	-4.307525	3.745190	-0.918223
N	0.614061	3.294436	-0.015706	H	-4.247282	2.175174	-0.130715
C	-2.282758	4.878562	-3.106536	H	-5.694580	2.643132	-1.032928
C	-1.489959	4.183703	-2.015567	H	-4.901949	0.078235	-6.281213
C	-0.440870	4.927303	-1.444886	H	-5.218420	-1.066341	-4.975657
C	0.611926	4.465641	-0.627959	H	-6.266219	0.339343	-5.183377
C	1.830191	5.361960	-0.528408	H	-0.382672	1.168885	-4.910647
C	-2.660839	2.212433	-2.567456	H	-0.334111	2.762097	-4.135673
C	-4.025322	2.080653	-2.271720	H	-0.060238	1.323619	-3.172261
C	-4.840421	1.378227	-3.161551	H	3.365891	2.820541	3.634695
C	-4.336249	0.790941	-4.320102	H	4.503393	0.896302	-0.023415
C	-2.970866	0.913958	-4.577769	H	0.055752	3.795518	2.507450
C	-2.123065	1.609065	-3.718652	H	1.229218	3.882774	3.829426
C	-4.602708	2.696757	-1.024775	H	1.271984	5.070567	2.519301
C	-5.228996	-0.002431	-5.240574	H	5.125553	0.093810	2.714102
C	-0.648272	1.720791	-4.005539	H	5.445515	1.620134	3.555152
C	1.797011	2.839666	0.627594	H	6.181573	1.302236	1.977793
C	2.035032	3.124391	1.980759	H	3.296786	1.238263	-2.006468
C	3.178641	2.593501	2.586359	H	2.247345	2.668224	-2.121114
C	4.074894	1.785551	1.891180	H	1.566179	1.111792	-1.672491
C	3.816306	1.520983	0.544673	C	1.565555	-2.821659	2.239143
C	2.696029	2.037865	-0.101446	C	1.740826	-0.551324	2.502766
C	1.096669	4.013730	2.752981	C	2.309298	-3.069477	3.371953
C	5.271818	1.171979	2.571952	H	1.177785	-3.646356	1.643539
C	2.445815	1.751420	-1.556901	C	2.494314	-0.690474	3.647088
H	-3.357881	4.770504	-2.937559	H	1.491680	0.439289	2.136698
H	-2.071055	4.428076	-4.081628	H	2.492159	-4.095420	3.662027
H	-2.036586	5.939721	-3.157159	H	2.823779	0.207868	4.149898
H	-0.339150	5.942931	-1.807109	N	1.278959	-1.589251	1.791920
H	1.641562	6.338124	-0.976349	C	2.814011	-1.980609	4.122045
H	2.671271	4.898119	-1.055265	N	3.570169	-2.164221	5.238217
H	2.145684	5.502161	0.508103	C	4.049878	-1.013880	5.982358
H	-5.902959	1.292044	-2.941001	H	3.221668	-0.417933	6.384561
H	-2.552964	0.452233	-5.470271	H	4.658858	-1.357408	6.817952

H	4.672364	-0.366416	5.353943
C	3.842081	-3.505159	5.719340
H	4.454850	-3.445491	6.618206
H	2.918146	-4.038699	5.973759
H	4.391740	-4.093643	4.975168
C	-2.307929	1.628260	1.643993
H	-2.800347	2.548221	1.981429
H	-3.091529	0.952657	1.276449
C	-1.742028	0.969694	2.831064
F	-0.218286	0.287398	-0.473252
F	-1.100640	-0.200306	2.523388
F	-2.626140	0.612146	3.811433
F	-0.809053	1.717582	3.483065

int2.log

Mg	0.409585	-1.262655	-0.329365
N	1.771485	-2.558596	-1.268890
N	-1.036571	-2.712391	-0.328913
C	2.661801	-4.731056	-1.979596
C	1.582836	-3.865802	-1.355291
C	0.418484	-4.541842	-0.927239
C	-0.828157	-3.996348	-0.551718
C	-1.989814	-4.962455	-0.437211
C	2.902195	-1.994381	-1.928915
C	4.104621	-1.758419	-1.243979
C	5.177600	-1.193188	-1.937392
C	5.085883	-0.837780	-3.279846
C	3.876910	-1.069831	-3.935207
C	2.784751	-1.641091	-3.286771
C	4.263425	-2.106903	0.213083
C	6.240852	-0.182864	-3.994362
C	1.491279	-1.876074	-4.021615
C	-2.352703	-2.226127	-0.100109
C	-2.842897	-2.082935	1.207016
C	-4.102588	-1.516059	1.391254
C	-4.874804	-1.070633	0.318833
C	-4.360932	-1.218341	-0.967220
C	-3.107888	-1.784316	-1.196384
C	-2.007027	-2.497081	2.388936
C	-6.215500	-0.421496	0.553129
C	-2.558952	-1.913284	-2.594115
H	3.633292	-4.544574	-1.512583
H	2.774239	-4.490918	-3.041668
H	2.422017	-5.791082	-1.888983
H	0.436563	-5.618358	-1.046711
H	-1.664729	-5.995899	-0.562801
H	-2.739676	-4.737471	-1.202652
H	-2.490199	-4.860561	0.530153
H	6.113901	-1.028825	-1.406602
H	3.780703	-0.800199	-4.985067
H	3.822642	-3.078969	0.450066
H	3.765691	-1.366928	0.848677
H	5.320866	-2.129771	0.491158
H	7.194244	-0.420105	-3.513867
H	6.298129	-0.504112	-5.038506
H	6.133981	0.908253	-3.994092
H	1.581367	-1.580822	-5.070288
H	1.188626	-2.928203	-3.987468
H	0.691403	-1.293114	-3.555846
H	-4.487096	-1.413161	2.404748
H	-4.939361	-0.870251	-1.817339
H	-2.593527	-2.469544	3.310865
H	-1.595350	-3.504335	2.268779
H	-1.156349	-1.818930	2.507738
H	-6.812049	-0.399687	-0.363121
H	-6.095959	0.615370	0.889124
H	-6.787922	-0.951295	1.320961
H	-3.251269	-1.484601	-3.322537

H	-2.375683	-2.959994	-2.861968
H	-1.597705	-1.396030	-2.685470
Mg	-0.484462	1.558007	-0.956478
N	-1.820298	2.773902	-1.859360
N	0.814104	3.054552	-0.533116
C	-2.539575	4.827545	-2.993179
C	-1.530422	4.033049	-2.188016
C	-0.344174	4.706477	-1.847726
C	0.737612	4.264251	-1.060127
C	1.865775	5.245513	-0.823720
C	-3.090416	2.256517	-2.251374
C	-4.199367	2.427948	-1.408695
C	-5.444121	1.975728	-1.839452
C	-5.606418	1.322104	-3.060292
C	-4.477725	1.113467	-3.850854
C	-3.215166	1.564745	-3.464685
C	-4.034997	3.090892	-0.066179
C	-6.955283	0.791818	-3.475521
C	-2.006774	1.335362	-4.335156
C	1.903754	2.698024	0.308099
C	1.836013	2.967754	1.684028
C	2.875771	2.528564	2.504327
C	3.949079	1.795067	2.000033
C	3.988695	1.539554	0.629386
C	2.988132	1.988312	-0.233151
C	0.636225	3.673991	2.259055
C	5.007187	1.234802	2.915329
C	3.067301	1.729426	-1.714736
H	-3.479151	4.930918	-2.442014
H	-2.781276	4.311998	-3.927395
H	-2.161813	5.822786	-3.227547
H	-0.263325	5.720195	-2.219407
H	1.694787	6.184919	-1.350028
H	2.815013	4.818049	-1.161373
H	1.979262	5.457644	0.243503
H	-6.309420	2.125887	-1.197680
H	-4.580008	0.587148	-4.797853
H	-3.655454	4.114353	-0.156150
H	-3.314948	2.543377	0.552532
H	-4.986586	3.126409	0.470520
H	-7.040981	0.721772	-4.563367
H	-7.122777	-0.213137	-3.068469
H	-7.764694	1.430458	-3.110614
H	-2.273017	0.762783	-5.227084
H	-1.557751	2.281336	-4.657648
H	-1.227511	0.782803	-3.798306
H	2.825579	2.738716	3.571238
H	4.819341	0.974555	0.211165
H	-0.277401	3.102515	2.060051
H	0.733621	3.793662	3.340376
H	0.488314	4.664361	1.816537
H	4.787432	0.186394	3.153919
H	5.055706	1.788334	3.857925
H	5.997606	1.265587	2.452039
H	4.028854	1.287691	-1.979623
H	2.937028	2.654707	-2.285834
H	2.286463	1.036166	-2.045478
C	1.558807	-2.765756	2.053642
C	1.507717	-0.517416	2.465757
C	2.098811	-3.038117	3.292883
H	1.342604	-3.573364	1.357932
C	2.059107	-0.678076	3.720025
H	1.237470	0.469457	2.109062
H	2.296244	-4.067865	3.560067
H	2.221544	0.203897	4.324087
N	1.270017	-1.529052	1.623259
C	2.386101	-1.971928	4.176370
N	2.946971	-2.179327	5.401236

C	3.204181	-1.050909	6.275340
H	2.278740	-0.525635	6.541992
H	3.668499	-1.408200	7.194155
H	3.888326	-0.332436	5.807875
C	3.237373	-3.527329	5.848106
H	3.686592	-3.486837	6.840059
H	2.328281	-4.138106	5.912192
H	3.945979	-4.027948	5.177455
C	-2.217191	0.990074	2.948318
H	-3.290603	0.984961	2.816609
H	-1.568386	0.677475	2.134176
C	-1.718366	1.357138	4.110632
F	0.260765	0.035528	-1.831255
F	-0.542729	0.338425	0.500895
F	-0.431714	1.397426	4.419364
F	-2.421834	1.743734	5.163815

hfc-134a.log

C	1.010665	0.533580	-0.001006
H	1.385397	1.060923	-0.882186
H	-0.082320	0.543099	0.016056
C	1.525132	1.240673	1.247422
F	1.455692	-0.759565	-0.019310
F	1.097257	0.654352	2.365916
F	1.075263	2.504637	1.243707
F	2.857413	1.277690	1.287639

TS1.log

Mg	0.009391	-1.220739	1.019406
N	1.189608	-2.490412	-0.162162
N	-1.426854	-2.743470	1.172632
C	2.022261	-4.598956	-1.098843
C	1.023640	-3.796199	-0.286573
C	-0.067163	-4.513502	0.247372
C	-1.261357	-3.999239	0.795615
C	-2.432197	-4.960781	0.862171
C	2.183904	-1.851973	-0.954045
C	3.479080	-1.626496	-0.463469
C	4.409127	-0.994853	-1.290233
C	4.080713	-0.544486	-2.566236
C	2.778319	-0.751982	-3.019563
C	1.825793	-1.403741	-2.238834
C	3.868003	-2.034824	0.933836
C	5.086574	0.196861	-3.409684
C	0.427838	-1.633453	-2.749900
C	-2.710676	-2.297406	1.591452
C	-3.112215	-2.411864	2.932475
C	-4.326160	-1.842145	3.319869
C	-5.144589	-1.162049	2.416777
C	-4.730426	-1.080459	1.087330
C	-3.531589	-1.647171	0.655761
C	-2.252072	-3.140345	3.931189
C	-6.451093	-0.552756	2.860490
C	-3.104180	-1.548256	-0.782521
H	3.044507	-4.411605	-0.757554
H	1.983462	-4.298316	-2.150923
H	1.817382	-5.668437	-1.036254
H	-0.067865	-5.577777	0.045158
H	-2.114865	-5.986427	0.669867
H	-3.169188	-4.680916	0.101077
H	-2.945749	-4.921131	1.824781
H	5.419365	-0.837597	-0.915673
H	2.496453	-0.404377	-4.011671
H	3.434427	-2.998040	1.215604
H	3.510001	-1.298024	1.662066
H	4.955395	-2.100160	1.030981
H	5.023686	1.277433	-3.233037
H	6.109310	-0.114399	-3.178275

H	4.914595	0.030420	-4.476946
H	0.308499	-1.234135	-3.760286
H	0.171192	-2.698173	-2.767564
H	-0.306484	-1.144989	-2.097652
H	-4.630836	-1.917621	4.361769
H	-5.352146	-0.559919	0.361385
H	-2.606215	-2.967962	4.949919
H	-2.255634	-4.221699	3.751398
H	-1.214229	-2.808458	3.866913
H	-6.402432	-0.221264	3.901693
H	-7.271224	-1.275705	2.783543
H	-6.715943	0.310153	2.242438
H	-3.941793	-1.267291	-1.421807
H	-2.678634	-2.488095	-1.145402
H	-2.328642	-0.778502	-0.903794
Mg	-0.322632	1.649403	0.388394
N	-1.474849	2.845399	-0.845202
N	0.878507	3.281198	0.837494
C	-2.217532	4.855336	-2.042431
C	-1.297856	4.143054	-1.069580
C	-0.279109	4.920977	-0.492376
C	0.774679	4.502846	0.341839
C	1.867403	5.515163	0.619771
C	-2.433591	2.148651	-1.635119
C	-3.751095	1.985592	-1.181555
C	-4.662173	1.309178	-1.991059
C	-4.289847	0.753426	-3.214078
C	-2.963803	0.889716	-3.621223
C	-2.026974	1.581412	-2.854419
C	-4.160739	2.513317	0.167244
C	-5.279607	-0.027147	-4.041267
C	-0.602166	1.734105	-3.320714
C	1.996371	2.921406	1.638491
C	1.977417	3.143923	3.023874
C	3.033054	2.654600	3.796697
C	4.087529	1.939610	3.232679
C	4.091343	1.749978	1.850200
C	3.069238	2.238253	1.039709
C	0.836371	3.895182	3.656442
C	5.182117	1.350604	4.085711
C	3.097789	2.031573	-0.450526
H	-3.265350	4.717352	-1.759986
H	-2.108311	4.438495	-3.048708
H	-2.001238	5.923323	-2.082830
H	-0.257475	5.961936	-0.790502
H	1.613610	6.496328	0.217017
H	2.801982	5.180727	0.156407
H	2.066221	5.612745	1.689562
H	-5.689984	1.201504	-1.648732
H	-2.648979	0.451407	-4.566107
H	-3.894871	3.567119	0.296145
H	-3.650966	1.951669	0.957587
H	-5.237493	2.404708	0.321458
H	-5.029806	0.011261	-5.105213
H	-5.290159	-1.082887	-3.744967
H	-6.295699	0.358033	-3.916818
H	-0.450165	1.235385	-4.281345
H	-0.323626	2.787470	-3.433505
H	0.100377	1.299131	-2.598912
H	3.017549	2.826460	4.871668
H	4.910931	1.203997	1.386645
H	-0.123317	3.518931	3.292747
H	0.854803	3.798809	4.743899
H	0.870388	4.962727	3.411215
H	5.084489	0.259136	4.137864
H	5.145985	1.741475	5.107363
H	6.173173	1.570674	3.677065
H	4.092199	1.727539	-0.781929

H 2.807933 2.938352 -0.989513
H 2.401112 1.236925 -0.750778
C 1.371498 -2.759818 3.259200
C 1.549142 -0.504937 3.598333
C 2.128633 -3.045361 4.376401
H 0.980271 -3.563087 2.637758
C 2.312185 -0.679706 4.733896
H 1.304534 0.495483 3.252624
H 2.317223 -4.080786 4.627268
H 2.648507 0.200618 5.263988
N 1.074633 -1.515047 2.860519
C 2.634332 -1.984471 5.162501
N 3.395127 -2.208085 6.272332
C 3.822219 -1.088849 7.090204
H 2.968178 -0.537191 7.503274
H 4.421322 -1.460995 7.920955
H 4.441232 -0.390459 6.515685
C 3.652587 -3.565078 6.712375
H 4.286807 -3.539987 7.598192
H 2.726339 -4.094407 6.970579
H 4.177926 -4.139926 5.941072
C -2.329323 1.130389 3.231528
H -2.484923 2.193149 3.079215
H -3.149119 0.483084 2.939125
C -1.775619 0.813526 4.576016
F -1.133266 0.716780 2.064219
F -1.375517 -0.454834 4.702987
F -2.715561 1.012672 5.540577
F -0.732385 1.592192 4.911196

TS2.log

Mg 0.153942 -1.202550 0.285262
N 1.369620 -2.404409 -0.945979
N -1.359338 -2.597138 0.204969
C 2.153367 -4.535039 -1.883316
C 1.159267 -3.704175 -1.092623
C 0.030964 -4.397724 -0.605772
C -1.192049 -3.857577 -0.151704
C -2.382835 -4.795183 -0.157564
C 2.393956 -1.812759 -1.741465
C 3.693741 -1.626680 -1.248358
C 4.655833 -1.054031 -2.084410
C 4.361328 -0.648955 -3.383367
C 3.058271 -0.833059 -3.846134
C 2.070326 -1.404878 -3.049262
C 4.070101 -2.042819 0.149795
C 5.403835 0.010790 -4.249939
C 0.669744 -1.592111 -3.568383
C -2.673742 -2.111102 0.460463
C -3.195805 -2.147058 1.761295
C -4.449951 -1.585864 1.996713
C -5.188250 -0.982888 0.978717
C -4.651872 -0.973993 -0.308159
C -3.403368 -1.529069 -0.587912
C -2.396660 -2.769233 2.876446
C -6.510103 -0.318025 1.270641
C -2.843576 -1.493679 -1.983874
H 3.176992 -4.360312 -1.540404
H 2.125050 -4.254263 -2.941128
H 1.928563 -5.599318 -1.804305
H 0.022374 -5.463185 -0.801238
H -2.071919 -5.829040 -0.312640
H -3.062666 -4.515195 -0.969576
H -2.957232 -4.728644 0.769331
H 5.668068 -0.925893 -1.704651
H 2.802326 -0.520519 -4.856541
H 3.659032 -3.022360 0.408494
H 3.684147 -1.330792 0.886939

H	5.157274	-2.082782	0.261628
H	6.415412	-0.259447	-3.934226
H	5.288496	-0.274221	-5.299762
H	5.322264	1.102669	-4.196971
H	0.577833	-1.215460	-4.590150
H	0.371037	-2.646211	-3.563242
H	-0.042010	-1.058326	-2.932845
H	-4.849329	-1.600264	3.008634
H	-5.210378	-0.517032	-1.122522
H	-2.882405	-2.607881	3.841698
H	-2.274212	-3.849026	2.733280
H	-1.391677	-2.338826	2.920859
H	-7.124627	-0.241245	0.369161
H	-6.359420	0.697855	1.654687
H	-7.079285	-0.869962	2.024470
H	-3.597346	-1.155482	-2.696460
H	-2.472159	-2.474207	-2.299072
H	-1.998336	-0.801326	-2.038924
Mg	-1.012014	1.888198	-0.285099
N	-1.841923	2.912399	-1.854626
N	0.503884	3.281138	-0.061194
C	-2.381309	4.902390	-3.175610
C	-1.576105	4.184150	-2.111266
C	-0.554987	4.927354	-1.486793
C	0.475868	4.472972	-0.639555
C	1.644098	5.417989	-0.440723
C	-2.728920	2.202527	-2.711734
C	-4.090453	2.070672	-2.410537
C	-4.903576	1.338792	-3.281997
C	-4.400433	0.727227	-4.424803
C	-3.035569	0.858135	-4.692913
C	-2.191446	1.578830	-3.855612
C	-4.697961	2.699578	-1.182273
C	-5.286077	-0.095238	-5.326293
C	-0.719487	1.699699	-4.151303
C	1.685650	2.853657	0.610836
C	1.921554	3.146561	1.961720
C	3.057261	2.609187	2.580027
C	3.962772	1.806902	1.894062
C	3.722961	1.556470	0.540880
C	2.606782	2.068933	-0.113453
C	1.021208	4.071429	2.739806
C	5.171272	1.212984	2.572650
C	2.402034	1.825330	-1.584342
H	-3.451647	4.830899	-2.959798
H	-2.225948	4.435538	-4.153443
H	-2.103306	5.954554	-3.243469
H	-0.466952	5.956407	-1.812640
H	1.492675	6.355276	-0.976678
H	2.565327	4.952415	-0.805948
H	1.798831	5.640358	0.618120
H	-5.964754	1.251072	-3.053819
H	-2.617961	0.379340	-5.576389
H	-4.012004	3.405680	-0.711194
H	-4.944386	1.930919	-0.440863
H	-5.625177	3.225021	-1.431803
H	-5.037325	0.060568	-6.380307
H	-5.169814	-1.165288	-5.118371
H	-6.341206	0.155177	-5.186354
H	-0.455467	1.141809	-5.053289
H	-0.417766	2.743176	-4.293493
H	-0.125929	1.311432	-3.318001
H	3.233059	2.836667	3.630372
H	4.426749	0.953089	-0.028386
H	0.040787	4.162917	2.270306
H	0.882360	3.716863	3.764051
H	1.460234	5.074735	2.799018
H	5.122337	0.117617	2.566244

H	5.246672	1.541589	3.613896
H	6.096035	1.501773	2.062589
H	3.276972	1.342754	-2.021376
H	2.210338	2.761033	-2.119773
H	1.540240	1.174581	-1.756350
C	1.594624	-2.923766	2.336701
C	1.869969	-0.704793	2.821211
C	2.446432	-3.308969	3.351050
H	1.126066	-3.671297	1.699898
C	2.747151	-0.981982	3.848715
H	1.611592	0.322279	2.589043
H	2.624750	-4.364498	3.507970
H	3.168485	-0.151758	4.399534
N	1.303505	-1.646013	2.053105
C	3.075979	-2.322489	4.143945
N	3.953766	-2.644679	5.135013
C	4.606574	-1.592699	5.890591
H	3.881683	-0.988666	6.449453
H	5.295068	-2.040336	6.606748
H	5.182597	-0.926824	5.236297
C	4.269211	-4.034466	5.401673
H	4.975147	-4.089019	6.229910
H	3.374648	-4.601837	5.684414
H	4.728463	-4.519372	4.531242
C	-2.491105	1.766978	1.392405
H	-2.734302	2.805533	1.610653
H	-3.339822	1.145191	1.114490
C	-1.694535	1.176758	2.395452
F	-0.287142	0.236421	-0.914244
F	-0.461339	0.374475	1.622954
F	-2.104314	0.158581	3.131980
F	-0.965070	1.965628	3.165394

TS3.log

Mg	0.032179	-1.275581	-0.077594
N	1.161514	-2.423122	-1.380826
N	-1.399089	-2.760121	0.012465
C	2.006018	-4.503351	-2.371540
C	1.016835	-3.736895	-1.514361
C	-0.030143	-4.490125	-0.951132
C	-1.221503	-4.011868	-0.359321
C	-2.360951	-5.000701	-0.226566
C	2.131296	-1.768208	-2.190381
C	3.441280	-1.556788	-1.731690
C	4.350347	-0.918847	-2.575424
C	3.986527	-0.446104	-3.834738
C	2.671771	-0.641603	-4.254809
C	1.739015	-1.303594	-3.457920
C	3.863335	-1.992437	-0.352226
C	4.972586	0.303858	-4.693580
C	0.331968	-1.541922	-3.941547
C	-2.683509	-2.314271	0.434559
C	-3.040281	-2.356515	1.790885
C	-4.258536	-1.802251	2.179011
C	-5.113961	-1.187698	1.262752
C	-4.738286	-1.169753	-0.079011
C	-3.536589	-1.731263	-0.512524
C	-2.098457	-2.946146	2.806748
C	-6.387679	-0.524702	1.723099
C	-3.149970	-1.691749	-1.967890
H	3.034955	-4.309224	-2.055275
H	1.933759	-4.180782	-3.415242
H	1.818779	-5.576675	-2.327651
H	-0.003881	-5.553459	-1.155994
H	-2.029952	-6.019405	-0.432281
H	-3.151431	-4.743196	-0.939925
H	-2.812314	-4.963499	0.767961
H	5.371901	-0.772053	-2.228699

H	2.364559	-0.279661	-5.233966	C	-1.827085	1.558943	-3.929228
H	3.474478	-2.981998	-0.096588	C	-4.206503	2.427528	-1.068988
H	3.483753	-1.297048	0.404731	C	-4.914028	-0.212229	-5.306708
H	4.953367	-2.014833	-0.268846	C	-0.383624	1.780749	-4.302330
H	4.950716	1.376333	-4.466182	C	1.982199	2.891440	0.608576
H	5.995300	-0.045959	-4.526339	C	2.038201	3.105596	1.994377
H	4.744515	0.190478	-5.757059	C	3.110668	2.563721	2.707304
H	0.193999	-1.154338	-4.953690	C	4.107463	1.813072	2.086904
H	0.081984	-2.608071	-3.944861	C	4.036947	1.635508	0.704485
H	-0.403177	-1.054464	-3.291489	C	2.993103	2.168685	-0.047925
H	-4.531546	-1.823242	3.231705	C	0.971994	3.910484	2.689660
H	-5.392303	-0.703973	-0.813642	C	5.214455	1.170714	2.883254
H	-2.559964	-2.974582	3.796125	C	2.932613	1.974639	-1.539789
H	-1.787885	-3.961657	2.539192	H	-3.180468	4.670551	-2.960524
H	-1.191046	-2.339211	2.876745	H	-1.945945	4.394802	-4.177218
H	-7.096771	-0.407224	0.898900	H	-1.913798	5.889519	-3.222907
H	-6.182219	0.472527	2.128457	H	-0.216471	5.949087	-1.860578
H	-6.876787	-1.102986	2.512493	H	1.678701	6.461021	-0.865487
H	-4.027428	-1.535457	-2.597634	H	2.826618	5.110312	-0.914963
H	-2.648732	-2.610696	-2.283584	H	2.121915	5.592676	0.619813
H	-2.462027	-0.861005	-2.169449	H	-5.538884	1.016719	-2.962302
Mg	-0.557999	1.823479	-0.371488	H	-2.282117	0.392403	-5.669362
N	-1.459532	2.841361	-1.894178	H	-3.949881	3.480958	-0.923345
N	0.846524	3.295130	-0.144050	H	-3.781442	1.881003	-0.220275
C	-2.119862	4.819541	-3.183602	H	-5.293363	2.323075	-1.019017
C	-1.255117	4.130597	-2.146829	H	-4.606699	-0.158235	-6.354840
C	-0.256766	4.914651	-1.542540	H	-4.881384	-1.267527	-5.009937
C	0.782710	4.498091	-0.687117	H	-5.955675	0.114395	-5.240787
C	1.908679	5.481164	-0.445919	H	-0.141037	1.277609	-5.241600
C	-2.338272	2.109536	-2.741599	H	-0.152538	2.845104	-4.417038
C	-3.675242	1.893962	-2.373389	H	0.292880	1.399512	-3.528841
C	-4.496327	1.168502	-3.236011	H	3.156311	2.726907	3.782680
C	-4.018852	0.621385	-4.425614	H	4.812085	1.065170	0.195885
C	-2.677591	0.819572	-4.749908	H	-0.026575	3.612202	2.363408

H	1.029239	3.783700	3.772538
H	1.075200	4.979375	2.469468
H	5.038282	0.092555	2.986039
H	5.282610	1.597476	3.888639
H	6.186320	1.298501	2.397022
H	3.899836	1.654794	-1.929115
H	2.629524	2.889630	-2.055897
H	2.206811	1.197449	-1.813375
C	1.472544	-2.828399	2.095408
C	1.566981	-0.572591	2.475644
C	2.223469	-3.106464	3.217457
H	1.112248	-3.632921	1.457436
C	2.316123	-0.741642	3.620474
H	1.290502	0.425578	2.148692
H	2.443375	-4.138733	3.455160
H	2.607922	0.140805	4.172484
N	1.140285	-1.586399	1.713158
C	2.679159	-2.041734	4.030091
N	3.430431	-2.257367	5.146760
C	3.800071	-1.137712	5.992776
H	2.918545	-0.628019	6.401455
H	4.400538	-1.502288	6.825798
H	4.400396	-0.405653	5.440931
C	3.722767	-3.612029	5.572979
H	4.341540	-3.579422	6.469316
H	2.809333	-4.172979	5.808822
H	4.278566	-4.159350	4.803124
C	-1.907824	0.790788	1.941371
H	-2.929976	0.476285	1.727369
H	-0.883548	0.245865	0.783425
C	-1.731110	1.027665	3.396025
F	-1.767064	2.113271	1.313300
F	-1.999918	-0.105147	4.072357
F	-2.518540	1.992271	3.956264
F	-0.467907	1.393283	3.727082

TS4.log

Mg	-0.119648	-1.142120	1.124305
N	1.094683	-2.407735	-0.038852
N	-1.531156	-2.675318	1.272875
C	1.967351	-4.520236	-0.931685
C	0.956094	-3.717898	-0.134824
C	-0.117639	-4.445018	0.422774
C	-1.323186	-3.943395	0.954432
C	-2.454340	-4.943925	1.099022
C	2.074084	-1.770855	-0.851214
C	3.365816	-1.506286	-0.371863
C	4.284319	-0.891849	-1.224504
C	3.948370	-0.500628	-2.517778
C	2.648028	-0.743674	-2.959053
C	1.705813	-1.375552	-2.150335
C	3.764186	-1.860106	1.037232
C	4.941789	0.216487	-3.396062
C	0.307476	-1.635894	-2.644772
C	-2.841522	-2.277058	1.658185
C	-3.232931	-2.326021	3.004428
C	-4.516926	-1.901345	3.348349
C	-5.408886	-1.411337	2.395758
C	-4.990102	-1.362716	1.067432
C	-3.720400	-1.786592	0.679740
C	-2.273604	-2.820493	4.057554
C	-6.769893	-0.900919	2.795770
C	-3.284516	-1.707760	-0.758471
H	2.987327	-4.301071	-0.602917
H	1.914953	-4.249447	-1.991226
H	1.786113	-5.591839	-0.838867
H	-0.089652	-5.515568	0.259568
H	-2.108254	-5.961679	0.915132

H	-3.243762	-4.711583	0.376132
H	-2.915313	-4.897719	2.088927
H	5.292890	-0.705715	-0.858862
H	2.358926	-0.438430	-3.962942
H	3.366574	-2.830869	1.345085
H	3.378456	-1.115198	1.742581
H	4.852989	-1.880841	1.139293
H	5.970945	-0.026186	-3.116463
H	4.806047	-0.044382	-4.449705
H	4.825138	1.303794	-3.311953
H	0.181151	-1.281565	-3.670989
H	0.055534	-2.701347	-2.614317
H	-0.426529	-1.120839	-2.012536
H	-4.826064	-1.951071	4.391339
H	-5.668321	-0.980441	0.306782
H	-2.734506	-2.798272	5.049325
H	-1.943638	-3.846624	3.862820
H	-1.367151	-2.206155	4.079831
H	-7.513241	-1.092869	2.016513
H	-6.744003	0.181689	2.964758
H	-7.118915	-1.371120	3.719633
H	-4.135203	-1.511994	-1.413784
H	-2.788831	-2.626380	-1.086471
H	-2.568367	-0.887089	-0.900198
Mg	-0.350720	1.713641	0.468191
N	-1.417187	2.900489	-0.827224
N	0.874003	3.306840	0.951027
C	-2.128475	4.921178	-2.014452
C	-1.259483	4.204708	-1.001047
C	-0.292914	4.981825	-0.335168
C	0.751158	4.546683	0.503167
C	1.822812	5.564213	0.838364
C	-2.301394	2.175255	-1.676174
C	-3.653518	2.004788	-1.347594

C	-4.455544	1.228860	-2.187347
C	-3.951020	0.607286	-3.324794
C	-2.598834	0.781610	-3.623810
C	-1.765064	1.554449	-2.820926
C	-4.252604	2.658447	-0.130138
C	-4.822146	-0.275562	-4.181915
C	-0.308889	1.734149	-3.163014
C	2.045908	2.910884	1.653449
C	2.121147	3.021441	3.050298
C	3.242083	2.506234	3.703513
C	4.261859	1.852528	3.014868
C	4.170491	1.770523	1.626025
C	3.086151	2.301165	0.930674
C	0.989100	3.634244	3.830535
C	5.405340	1.199432	3.747841
C	3.005072	2.195292	-0.568329
H	-3.187662	4.799001	-1.770880
H	-1.981775	4.491261	-3.010534
H	-1.898394	5.986338	-2.055182
H	-0.285545	6.035626	-0.585285
H	1.547553	6.561059	0.492069
H	2.764787	5.277819	0.357941
H	2.018022	5.603318	1.913056
H	-5.505681	1.099418	-1.930808
H	-2.181828	0.301564	-4.507034
H	-4.768986	3.589025	-0.396273
H	-3.486350	2.908620	0.604774
H	-4.982318	1.998056	0.345433
H	-4.703238	-1.329749	-3.904102
H	-5.880179	-0.022772	-4.069980
H	-4.562271	-0.187071	-5.240913
H	-0.070927	1.260691	-4.119165
H	-0.034720	2.792412	-3.223236
H	0.337340	1.283234	-2.398801

H	3.303723	2.599381	4.786843
H	4.958173	1.271639	1.064810
H	0.078960	3.039037	3.718141
H	1.230893	3.685901	4.895172
H	0.746418	4.643435	3.483951
H	5.231380	0.119582	3.841071
H	5.521389	1.609412	4.756116
H	6.353673	1.332368	3.218836
H	3.979349	1.946722	-0.993184
H	2.646689	3.123084	-1.023322
H	2.311239	1.395819	-0.862927
C	1.262339	-2.716883	3.331444
C	1.499948	-0.469494	3.680384
C	2.139357	-3.026127	4.349987
H	0.799809	-3.507889	2.745246
C	2.386588	-0.668677	4.718680
H	1.233303	0.537486	3.375531
H	2.347682	-4.066980	4.559711
H	2.798707	0.199466	5.213907
N	0.936404	-1.463052	2.983348
C	2.746592	-1.982156	5.085126
N	3.634984	-2.229051	6.090022
C	4.181913	-1.125773	6.856454
H	3.394712	-0.561015	7.371473
H	4.866262	-1.517217	7.608766
H	4.742792	-0.434189	6.217234
C	3.960848	-3.594630	6.450558
H	4.699434	-3.587014	7.251774
H	3.079912	-4.144808	6.805139
H	4.391948	-4.138872	5.602169
C	-2.501315	1.447523	3.184687
C	-2.311413	0.827196	4.536101
F	-1.325073	0.766878	1.981953
F	-2.287582	2.764607	3.152216
F	-3.702893	1.193319	2.691966
H	-2.593368	-0.224598	4.472515
H	-2.932306	1.343309	5.283183
F	-0.997503	0.936202	4.937696

hfc125.log

C	1.013173	0.535107	-0.006497
H	1.374857	1.047312	-0.905086
C	1.519732	1.251796	1.266042
F	1.456830	-0.736394	-0.001062
F	1.093972	0.651101	2.371658
F	1.086881	2.516133	1.268881
F	2.855628	1.264520	1.269627
F	-0.333375	0.528051	-0.001315

hfc143a.log

C	1.010246	0.537981	0.000869
H	1.375153	1.054078	-0.888568
H	-0.080902	0.539305	0.003023
C	1.511697	1.246378	1.228799
F	1.089675	0.649525	2.360141
F	1.089400	2.525275	1.277730
F	2.857465	1.274681	1.277577
H	1.374897	-0.490361	0.002650

hfc152a.log

C	1.011617	0.539636	0.002910
H	1.381692	1.063764	-0.881536
H	-0.080847	0.535216	-0.011575
C	1.503802	1.234422	1.243041
F	1.076510	2.534432	1.260384
F	2.871182	1.265394	1.259455

H	1.371798	-0.491758	-0.012392
H	1.176393	0.771569	2.181008

TS10.log

Mg	0.105886	-1.306862	0.905026
N	1.179919	-2.604101	-0.395478
N	-1.408571	-2.793410	1.170851
C	1.904806	-4.791115	-1.262839
C	0.975456	-3.916334	-0.442241
C	-0.092264	-4.586752	0.190622
C	-1.251181	-4.060519	0.804487
C	-2.395168	-5.042993	0.972530
C	2.120369	-2.044902	-1.310014
C	3.444411	-1.767685	-0.921904
C	4.319419	-1.200224	-1.853695
C	3.922197	-0.886604	-3.153396
C	2.602681	-1.168232	-3.515042
C	1.696021	-1.740581	-2.621158
C	3.928009	-2.079804	0.468127
C	4.874961	-0.245511	-4.128544
C	0.284189	-2.035028	-3.050030
C	-2.670535	-2.399467	1.705337
C	-2.961513	-2.566448	3.075401
C	-4.193358	-2.120356	3.565544
C	-5.145898	-1.510984	2.744522
C	-4.830695	-1.351542	1.392728
C	-3.615356	-1.788157	0.856975
C	-1.977317	-3.220580	4.006984
C	-6.480171	-1.073989	3.291813
C	-3.317404	-1.606035	-0.603970
H	2.953125	-4.576239	-1.035064
H	1.775038	-4.600418	-2.333424
H	1.712656	-5.850015	-1.080009
H	-0.110022	-5.659130	0.033379
H	-2.072205	-6.064091	0.762706
H	-3.200091	-4.791334	0.272156
H	-2.833695	-5.008660	1.972378
H	5.346254	-1.002222	-1.549887
H	2.266923	-0.940174	-4.525453
H	3.684359	-3.104855	0.766285
H	3.460676	-1.421237	1.207678
H	5.011983	-1.952140	0.540121
H	4.749112	0.844200	-4.151424
H	5.916849	-0.447185	-3.861771
H	4.711753	-0.610114	-5.147892
H	0.111222	-1.702818	-4.077082
H	0.052025	-3.105022	-2.999552
H	-0.438082	-1.530963	-2.398253
H	-4.411295	-2.254951	4.623771
H	-5.552278	-0.879963	0.728088
H	-2.334733	-3.178753	5.039170
H	-1.810202	-4.274243	3.754090
H	-1.003431	-2.728370	3.961664
H	-7.215386	-1.886959	3.239956
H	-6.889367	-0.231192	2.726489
H	-6.405035	-0.772127	4.341027
H	-4.172405	-1.177424	-1.130754
H	-3.048464	-2.552904	-1.084405
H	-2.467812	-0.925647	-0.750551
Mg	-0.270701	1.732758	0.459455
N	-1.319366	2.897130	-0.957780
N	0.981142	3.365868	0.925304
C	-1.806807	4.871838	-2.347567
C	-1.017419	4.157860	-1.266958
C	0.020486	4.906138	-0.681664
C	0.959474	4.540356	0.299534
C	2.022539	5.575414	0.612216

C	-2.331055	2.258681	-1.736405	H	-0.139469	2.569899	-3.534236
C	-3.678162	2.292498	-1.330241	H	0.116365	1.087158	-2.629388
C	-4.640014	1.658393	-2.123020	H	2.551602	3.671313	5.234845
C	-4.307052	0.985288	-3.299144	H	5.047995	1.755861	2.324211
C	-2.962123	0.959215	-3.676587	H	-0.389620	3.874868	3.179221
C	-1.967855	1.582536	-2.919721	H	0.372366	4.381563	4.697274
C	-4.087184	3.028862	-0.083885	H	0.525893	5.369793	3.241499
C	-5.357803	0.291591	-4.126964	H	5.458782	1.484065	4.855889
C	-0.533941	1.559965	-3.375001	H	4.580468	2.553007	5.966105
C	1.976331	3.158285	1.923344	H	5.789840	3.214039	4.866349
C	1.751200	3.586444	3.246992	H	4.426796	1.602899	0.081031
C	2.729918	3.334693	4.214670	H	3.349926	2.870352	-0.532522
C	3.925109	2.682496	3.908521	H	2.701395	1.284209	-0.141062
C	4.122687	2.264795	2.589608	C	1.553901	-2.895347	3.110831
C	3.172804	2.488982	1.590777	C	1.649455	-0.657073	3.578562
C	0.499543	4.337731	3.612860	C	2.253241	-3.222835	4.256512
C	4.989255	2.468868	4.954286	H	1.216012	-3.678822	2.435439
C	3.430067	2.040709	0.178461	C	2.351382	-0.876029	4.749160
H	-2.878390	4.860876	-2.127207	H	1.397235	0.356074	3.275990
H	-1.685844	4.373963	-3.315246	H	2.453367	-4.266594	4.464302
H	-1.483307	5.908790	-2.450630	H	2.633696	-0.018971	5.346457
H	0.135435	5.910235	-1.072109	N	1.240417	-1.636568	2.756192
H	1.878966	6.479813	0.018893	C	2.679455	-2.196484	5.133380
H	3.019143	5.174808	0.397761	N	3.365765	-2.466274	6.280749
H	2.021084	5.850548	1.670618	C	3.715459	-1.388099	7.186216
H	-5.682757	1.701989	-1.812018	H	2.825629	-0.868914	7.565792
H	-2.676291	0.447217	-4.594097	H	4.257348	-1.799380	8.038149
H	-3.889712	4.104556	-0.161490	H	4.364295	-0.650355	6.699266
H	-3.533368	2.679480	0.790992	C	3.642317	-3.839173	6.656706
H	-5.154983	2.899412	0.113427	H	4.223276	-3.846471	7.579212
H	-5.171192	0.417085	-5.198560	H	2.721352	-4.412162	6.829804
H	-5.376798	-0.787307	-3.928152	H	4.230511	-4.353660	5.887171
H	-6.357556	0.680689	-3.912142	C	-2.464689	1.180875	3.331158
H	-0.431493	1.006661	-4.312208	H	-3.204891	0.443863	3.032758

C	-1.869773	0.993681	4.699235
F	-1.226310	0.891003	2.147068
F	-1.446374	-0.260450	4.869304
F	-2.794775	1.235585	5.669028
F	-0.847795	1.822789	4.944225
F	-2.851429	2.445615	3.137193

TS11.log

Mg	0.027382	-1.376280	-0.143739
N	1.137101	-2.508970	-1.539496
N	-1.427222	-2.893260	0.027856
C	2.009483	-4.644500	-2.407289
C	1.030517	-3.838204	-1.574471
C	0.033977	-4.586373	-0.919364
C	-1.165080	-4.149752	-0.307608
C	-2.212120	-5.224568	-0.090003
C	2.039170	-1.906803	-2.466079
C	3.357354	-1.582786	-2.092998
C	4.207263	-1.004519	-3.041158
C	3.789239	-0.721395	-4.341529
C	2.475734	-1.047400	-4.687038
C	1.594651	-1.636310	-3.777745
C	3.861591	-1.864910	-0.703532
C	4.714519	-0.067897	-5.334561
C	0.195675	-1.998473	-4.198344
C	-2.742735	-2.599875	0.509073
C	-3.046425	-2.706428	1.879689
C	-4.343038	-2.413053	2.307430
C	-5.342523	-2.004352	1.423292
C	-5.013053	-1.899695	0.071712
C	-3.730979	-2.185719	-0.402885
C	-2.002813	-3.128055	2.878835
C	-6.720885	-1.653527	1.918919
C	-3.415113	-2.046515	-1.867152
H	3.044021	-4.359929	-2.193955
H	1.853045	-4.463280	-3.475969
H	1.892080	-5.713831	-2.223447
H	0.108536	-5.658903	-1.056533
H	-1.807542	-6.216408	-0.298646
H	-3.065032	-5.055483	-0.756611
H	-2.609550	-5.207988	0.928087
H	5.231264	-0.774425	-2.751010
H	2.126048	-0.847186	-5.698517
H	3.663592	-2.897228	-0.397103
H	3.372716	-1.223472	0.037473
H	4.939463	-1.690957	-0.638911
H	5.763917	-0.253646	-5.086480
H	4.537929	-0.436357	-6.350101
H	4.572142	1.019764	-5.356063
H	0.013932	-1.707760	-5.236359
H	0.007675	-3.074659	-4.112957
H	-0.552982	-1.505358	-3.569343
H	-4.573416	-2.500581	3.367754
H	-5.773674	-1.582881	-0.639769
H	-2.439246	-3.220634	3.876906
H	-1.542536	-4.088140	2.619522
H	-1.193394	-2.394366	2.935315
H	-7.007599	-2.268004	2.778312
H	-7.475367	-1.791416	1.138214
H	-6.769311	-0.605405	2.238975
H	-4.314547	-1.804844	-2.438424
H	-2.976914	-2.959529	-2.284535
H	-2.692889	-1.239683	-2.040259
Mg	-0.271468	1.902691	-0.527833
N	-1.192650	2.956879	-2.061906
N	1.153843	3.391335	-0.204178
C	-1.629557	4.915328	-3.484388

C	-0.843410	4.196185	-2.406621	H	-5.680469	1.034042	-5.917432
C	0.230879	4.916833	-1.850796	H	-5.013216	-0.554131	-5.540804
C	1.169475	4.544014	-0.870623	H	-6.234589	0.169040	-4.482920
C	2.275149	5.544516	-0.600466	H	-0.417999	0.985663	-5.393402
C	-2.237938	2.328839	-2.806582	H	-0.088426	2.568209	-4.674871
C	-3.572331	2.386806	-2.361804	H	0.177480	1.113661	-3.732309
C	-4.558900	1.740083	-3.112851	H	2.868148	3.673799	4.059314
C	-4.265303	1.039247	-4.282985	H	5.174691	1.608093	1.096269
C	-2.932182	0.990067	-4.697356	H	-0.118709	4.044362	2.086298
C	-1.912867	1.623145	-3.983013	H	0.708894	4.465588	3.593967
C	-3.945761	3.138329	-1.111873	H	0.900493	5.474243	2.158159
C	-5.353749	0.385690	-5.094572	H	5.604278	1.311583	3.651162
C	-0.490527	1.569156	-4.472046	H	4.862649	2.496170	4.742908
C	2.164424	3.160161	0.775340	H	6.084556	3.004694	3.577079
C	2.001431	3.613940	2.099645	H	4.467115	1.458311	-1.122920
C	2.997551	3.319091	3.038010	H	3.470801	2.799413	-1.711769
C	4.147157	2.602623	2.705054	H	2.723268	1.263842	-1.310881
C	4.284796	2.165171	1.384866	C	1.532353	-2.936971	2.046913
C	3.316398	2.430091	0.415224	C	1.560941	-0.691860	2.491417
C	0.808584	4.437491	2.506023	C	2.222539	-3.232171	3.206448
C	5.226161	2.337245	3.723068	H	1.226399	-3.736184	1.374895
C	3.507183	1.965695	-1.001997	C	2.248450	-0.878067	3.675213
H	-2.691375	4.963991	-3.223467	H	1.285698	0.311052	2.177391
H	-1.570415	4.382975	-4.438990	H	2.448429	-4.267577	3.428689
H	-1.259524	5.931173	-3.630664	H	2.495500	-0.007202	4.267819
H	0.378282	5.905671	-2.268142	N	1.189940	-1.690997	1.673832
H	2.163326	6.433879	-1.222343	C	2.604050	-2.185563	4.080139
H	3.254022	5.098341	-0.805829	N	3.273050	-2.423996	5.243537
H	2.289150	5.853630	0.448459	C	3.574449	-1.326691	6.144151
H	-5.590589	1.792944	-2.769409	H	2.663120	-0.825379	6.495128
H	-2.675703	0.449249	-5.606921	H	4.104672	-1.714850	7.014003
H	-3.513108	4.143910	-1.094443	H	4.217422	-0.579487	5.663965
H	-3.590246	2.626575	-0.212808	C	3.577140	-3.785067	5.641535
H	-5.031943	3.233470	-1.029416	H	4.135512	-3.766764	6.577640

H	2.667657	-4.380214	5.799116
H	4.197337	-4.291684	4.892174
C	-1.742506	0.969656	1.825242
H	-0.763533	0.322986	0.737207
C	-1.775578	1.245071	3.322303
F	-1.455840	2.267703	1.245297
F	-2.119850	0.129150	3.983174
F	-2.635508	2.212184	3.723932
F	-0.555267	1.617773	3.754332
F	-3.009884	0.708002	1.422618

TS12.log

Mg	0.051673	-1.276261	0.945668
N	1.167898	-2.539272	-0.347820
N	-1.398298	-2.808190	1.251372
C	1.957883	-4.708403	-1.204907
C	1.012902	-3.858778	-0.376098
C	-0.014327	-4.562157	0.287552
C	-1.181399	-4.077314	0.918178
C	-2.260335	-5.115963	1.163258
C	2.096112	-1.964278	-1.265974
C	3.418903	-1.676036	-0.881737
C	4.289550	-1.113817	-1.820672
C	3.887597	-0.812132	-3.121828
C	2.567160	-1.098117	-3.476722
C	1.665192	-1.668222	-2.576440
C	3.904657	-1.970661	0.511436
C	4.836304	-0.179005	-4.106148
C	0.253741	-1.972654	-2.999499
C	-2.681323	-2.482436	1.786261
C	-2.947317	-2.617209	3.164180
C	-4.225386	-2.305472	3.641988
C	-5.242695	-1.848328	2.801648
C	-4.948261	-1.708378	1.444449
C	-3.689533	-2.012750	0.921035
C	-1.887691	-3.092786	4.123537
C	-6.601513	-1.491556	3.344949
C	-3.414956	-1.843549	-0.547217
H	3.002538	-4.463897	-0.990944
H	1.809557	-4.522906	-2.274015
H	1.797890	-5.771834	-1.018103
H	0.012871	-5.637010	0.150970
H	-1.902678	-6.118862	0.924644
H	-3.131371	-4.904604	0.532953
H	-2.618514	-5.105161	2.196009
H	5.316589	-0.910144	-1.521334
H	2.227561	-0.877913	-4.487555
H	3.672412	-2.995583	0.819145
H	3.429870	-1.308994	1.243487
H	4.987013	-1.830281	0.583153
H	5.879434	-0.361510	-3.830756
H	4.682567	-0.567165	-5.118276
H	4.696160	0.908142	-4.152872
H	0.077796	-1.652890	-4.030006
H	0.026558	-3.043007	-2.935757
H	-0.470528	-1.463426	-2.353710
H	-4.430745	-2.432867	4.704118
H	-5.721448	-1.352477	0.765977
H	-2.298617	-3.201884	5.131399
H	-1.462390	-4.057727	3.827856
H	-1.049409	-2.390453	4.171182
H	-7.391888	-1.708426	2.619510
H	-6.664419	-0.422979	3.584177
H	-6.824355	-2.044279	4.262672
H	-4.312815	-1.521254	-1.079574
H	-3.052784	-2.770111	-1.005779
H	-2.641301	-1.083508	-0.715969

Mg	-0.338207	1.793924	0.500080	H	-2.465542	0.518942	-4.600052
N	-1.326146	2.992286	-0.911671	H	-4.500643	4.138262	-0.492263
N	0.988489	3.357128	0.978733	H	-3.406021	3.243237	0.555242
C	-1.832082	5.034874	-2.185598	H	-5.037110	2.626281	0.237977
C	-1.053138	4.276606	-1.129516	H	-5.074309	0.601439	-5.299188
C	-0.050338	5.008648	-0.465555	H	-4.996108	-0.795111	-4.227607
C	0.921630	4.581137	0.458154	H	-6.186959	0.476608	-3.930102
C	1.974473	5.608370	0.826064	H	-0.260285	1.175742	-4.244305
C	-2.299540	2.359241	-1.742214	H	-0.030733	2.718969	-3.405349
C	-3.664670	2.367693	-1.403661	H	0.212629	1.210220	-2.539256
C	-4.570330	1.690051	-2.229285	H	3.071211	3.160336	5.080471
C	-4.167789	1.014996	-3.379277	H	5.220841	1.724902	1.662360
C	-2.806395	1.029190	-3.700466	H	-0.091552	3.303663	3.638087
C	-1.866607	1.684440	-2.905729	H	0.914821	4.001076	4.914779
C	-4.175201	3.128291	-0.209474	H	0.591999	4.902303	3.426745
C	-5.158609	0.289113	-4.252144	H	5.530669	0.940791	4.494060
C	-0.413540	1.698096	-3.296230	H	5.361802	2.478998	5.352766
C	2.092809	3.050826	1.826791	H	6.400370	2.363456	3.926476
C	2.023643	3.288139	3.213448	H	4.320046	1.826074	-0.486857
C	3.123937	2.955404	4.011968	H	3.176773	3.147794	-0.790516
C	4.283439	2.389351	3.481348	H	2.582949	1.510921	-0.543522
C	4.324820	2.158079	2.103936	C	1.535535	-2.798019	3.170606
C	3.254168	2.477593	1.266750	C	1.536584	-0.557346	3.637697
C	0.799142	3.905454	3.831695	C	2.220046	-3.098459	4.332265
C	5.452474	2.027171	4.360263	H	1.242630	-3.593890	2.488841
C	3.342548	2.230976	-0.214109	C	2.216716	-0.749839	4.825651
H	-2.902050	5.040634	-1.957858	H	1.257271	0.445861	3.327046
H	-1.724876	4.556671	-3.164721	H	2.457289	-4.133570	4.544363
H	-1.489150	6.067709	-2.264166	H	2.457159	0.117794	5.425950
H	0.031135	6.046414	-0.766135	N	1.182001	-1.551812	2.807915
H	1.756624	6.576282	0.371929	C	2.583737	-2.057133	5.219734
H	2.962894	5.282212	0.484368	N	3.248107	-2.300858	6.385200
H	2.049487	5.739168	1.909301	C	3.548443	-1.207820	7.290741
H	-5.625870	1.701652	-1.961471	H	2.636460	-0.703297	7.635040

H	4.067715	-1.601077	8.164908
H	4.199300	-0.461436	6.819179
C	3.586447	-3.660456	6.759215
H	4.134697	-3.645619	7.701364
H	2.692031	-4.282603	6.896664
H	4.226714	-4.136392	6.006277
C	-2.700912	1.658700	3.206530
C	-2.530308	0.859114	4.469172
F	-1.436191	1.060940	2.038427
F	-2.518311	2.964168	3.335332
F	-3.845784	1.413491	2.598715
H	-2.687574	-0.203645	4.268977
F	-1.300198	1.068884	4.993786
F	-3.438308	1.258214	5.425992

TS5.log

Mg	-0.068231	-1.237743	1.005597
N	1.096851	-2.544910	-0.227549
N	-1.525721	-2.777842	1.284245
C	1.871213	-4.721528	-1.081100
C	0.925317	-3.860155	-0.264945
C	-0.127581	-4.551181	0.373455
C	-1.307007	-4.051357	0.966629
C	-2.400512	-5.081217	1.186483
C	2.068697	-1.980073	-1.105518
C	3.392818	-1.755133	-0.685278
C	4.306830	-1.203464	-1.589268
C	3.948147	-0.852107	-2.890125
C	2.625293	-1.074001	-3.281100
C	1.681179	-1.630868	-2.416969
C	3.838021	-2.100100	0.710159
C	4.944758	-0.238376	-3.838991
C	0.267723	-1.860287	-2.876644
C	-2.830339	-2.443197	1.760538
C	-3.149229	-2.545968	3.129009
C	-4.443225	-2.227041	3.550790
C	-5.428791	-1.797314	2.661629
C	-5.085138	-1.691697	1.312976
C	-3.806421	-2.002201	0.845340
C	-2.128863	-3.015472	4.132129
C	-6.806990	-1.427605	3.144298
C	-3.479627	-1.878724	-0.617293
H	2.916325	-4.505258	-0.841029
H	1.750980	-4.516801	-2.150433
H	1.682133	-5.783442	-0.913403
H	-0.110052	-5.626411	0.237728
H	-2.036086	-6.090255	0.986504
H	-3.241621	-4.882375	0.512962
H	-2.805171	-5.045719	2.201213
H	5.333988	-1.049479	-1.261609
H	2.317672	-0.814234	-4.292843
H	3.508428	-3.098871	1.012915
H	3.421694	-1.397400	1.439587
H	4.928358	-2.060104	0.790389
H	5.972664	-0.411382	-3.506297
H	4.844672	-0.651035	-4.848504
H	4.804718	0.846723	-3.920057
H	0.139395	-1.549267	-3.916901
H	-0.025694	-2.913042	-2.797801
H	-0.439024	-1.291946	-2.259928
H	-4.686209	-2.322499	4.608278
H	-5.834601	-1.358937	0.596871
H	-2.539138	-2.987078	5.146059
H	-1.798745	-4.042847	3.937715
H	-1.227515	-2.396070	4.104873
H	-7.570961	-1.665822	2.397350
H	-6.879422	-0.352489	3.350416

H	-7.063686	-1.954730	4.068527
H	-4.354744	-1.559925	-1.188481
H	-3.117738	-2.823756	-1.037415
H	-2.688574	-1.136959	-0.783245
Mg	-0.317761	1.751654	0.440597
N	-1.367386	2.954414	-0.922954
N	0.950657	3.361311	0.959450
C	-1.985984	5.020444	-2.108008
C	-1.165108	4.259219	-1.085471
C	-0.195056	5.015271	-0.397768
C	0.817959	4.598770	0.484868
C	1.841837	5.655107	0.853247
C	-2.287701	2.308101	-1.802574
C	-3.647925	2.191489	-1.460725
C	-4.513377	1.543155	-2.347230
C	-4.071337	0.997222	-3.552696
C	-2.715187	1.118494	-3.864618
C	-1.814645	1.762218	-3.013644
C	-4.170586	2.759629	-0.169733
C	-5.018702	0.281494	-4.480426
C	-0.362483	1.882788	-3.389158
C	2.083337	3.071009	1.772743
C	2.050419	3.284412	3.164904
C	3.178914	2.960275	3.928481
C	4.334684	2.429201	3.357116
C	4.341017	2.219822	1.974966
C	3.241607	2.528597	1.173143
C	0.840577	3.883778	3.829145
C	5.540344	2.087462	4.193871
C	3.293339	2.300823	-0.312154
H	-3.058084	4.885204	-1.937828
H	-1.784424	4.649336	-3.118574
H	-1.758833	6.087425	-2.081131
H	-0.170989	6.067427	-0.655690
H	1.569266	6.628722	0.442850
H	2.827510	5.380622	0.461134
H	1.955408	5.752005	1.936305
H	-5.567311	1.468269	-2.084035
H	-2.344892	0.706373	-4.802076
H	-3.917131	3.818488	-0.051512
H	-3.745822	2.238237	0.692317
H	-5.258446	2.662638	-0.114587
H	-4.950654	-0.806934	-4.359809
H	-6.057595	0.566631	-4.289215
H	-4.796822	0.502873	-5.529629
H	-0.176475	1.440816	-4.371891
H	-0.031114	2.926801	-3.418248
H	0.280040	1.371061	-2.661849
H	3.151941	3.146840	5.001647
H	5.232097	1.810558	1.501799
H	-0.085496	3.460347	3.436080
H	0.871356	3.728380	4.911946
H	0.777490	4.966156	3.660519
H	5.718831	1.005253	4.217522
H	5.420927	2.430043	5.226289
H	6.449666	2.550394	3.794549
H	4.280487	1.947744	-0.620066
H	3.061429	3.213487	-0.871643
H	2.560784	1.544238	-0.621756
C	1.338082	-2.790018	3.279639
C	1.571458	-0.540009	3.618385
C	2.093178	-3.094110	4.396644
H	0.929623	-3.587877	2.662795
C	2.334349	-0.733688	4.755633
H	1.364070	0.467499	3.265201
H	2.265098	-4.134843	4.641697
H	2.711073	0.136660	5.277393
N	1.061551	-1.537027	2.878834

C	2.620750	-2.048614	5.190833
N	3.364449	-2.295058	6.307523
C	3.881878	-1.191590	7.093285
H	3.073861	-0.554617	7.474870
H	4.428769	-1.587946	7.949049
H	4.569875	-0.564870	6.511676
C	3.651888	-3.660324	6.703008
H	4.258449	-3.649656	7.608795
H	2.733878	-4.222025	6.918357
H	4.212421	-4.197964	5.927483
C	-2.439745	1.478561	3.333296
C	-2.011599	0.953560	4.652238
F	-1.254345	0.856576	1.999029
F	-2.385061	2.810761	3.213814
F	-3.633408	1.068941	2.927648
H	-2.058075	-0.135119	4.634640
H	-2.674628	1.326711	5.449179
H	-0.989824	1.270428	4.863327

TS6.log

Mg	0.112680	-1.362019	-0.143625
N	1.198580	-2.561276	-1.521133
N	-1.380290	-2.860463	0.022907
C	2.003675	-4.716216	-2.403096
C	1.039120	-3.883307	-1.579066
C	-0.001473	-4.599306	-0.956112
C	-1.185482	-4.117638	-0.348320
C	-2.297632	-5.136015	-0.188977
C	2.121933	-1.972551	-2.434068
C	3.438747	-1.661942	-2.044560
C	4.301947	-1.076049	-2.976036
C	3.900207	-0.774389	-4.277265
C	2.589095	-1.089913	-4.641161
C	1.694890	-1.683899	-3.748387
C	3.928511	-1.964574	-0.654525
C	4.839659	-0.113215	-5.251837
C	0.297024	-2.027634	-4.187598
C	-2.669407	-2.493327	0.517615
C	-2.990817	-2.646151	1.881348
C	-4.251097	-2.241732	2.325949
C	-5.199250	-1.680166	1.468107
C	-4.856160	-1.538680	0.123122
C	-3.608942	-1.934688	-0.368805
C	-2.001289	-3.226684	2.854999
C	-6.536219	-1.219586	1.987574
C	-3.277544	-1.765319	-1.825596
H	3.042883	-4.469743	-2.165523
H	1.875763	-4.519658	-3.472800
H	1.845852	-5.782715	-2.232603
H	0.023546	-5.670873	-1.117970
H	-1.944008	-6.144602	-0.410019
H	-3.114624	-4.902064	-0.881061
H	-2.729941	-5.121882	0.814447
H	5.323979	-0.855421	-2.671541
H	2.251399	-0.875553	-5.653953
H	3.716505	-2.998082	-0.361774
H	3.439447	-1.326791	0.089277
H	5.007732	-1.803132	-0.579270
H	4.696459	0.974488	-5.269090
H	5.885402	-0.299272	-4.988900
H	4.679451	-0.475510	-6.272396
H	0.123919	-1.709987	-5.219321
H	0.101246	-3.104402	-4.131134
H	-0.451385	-1.546561	-3.549113
H	-4.492722	-2.360871	3.380667
H	-5.577498	-1.109684	-0.570217
H	-2.436591	-3.288663	3.855970

H	-1.673975	-4.231893	2.566186
H	-1.104055	-2.605257	2.916255
H	-6.948753	-1.925488	2.716064
H	-7.263418	-1.107985	1.177742
H	-6.453010	-0.248709	2.491273
H	-4.132604	-1.370046	-2.378271
H	-2.970274	-2.709100	-2.289373
H	-2.447011	-1.061861	-1.963147
Mg	-0.275050	1.821896	-0.552670
N	-1.318945	2.906696	-1.985295
N	1.032981	3.380350	-0.124728
C	-1.784450	4.867419	-3.398825
C	-0.999026	4.159927	-2.312338
C	0.051084	4.903126	-1.742042
C	1.004307	4.544833	-0.770118
C	2.072984	5.580502	-0.485518
C	-2.333971	2.265458	-2.761451
C	-3.687929	2.330333	-2.382029
C	-4.641353	1.690600	-3.181313
C	-4.296309	0.987769	-4.335955
C	-2.945825	0.939011	-4.690248
C	-1.958904	1.565615	-3.927270
C	-4.125655	3.095168	-1.161013
C	-5.341380	0.292242	-5.169117
C	-0.518578	1.525971	-4.361463
C	2.058661	3.171133	0.846084
C	1.919942	3.650278	2.162885
C	2.932096	3.372593	3.089513
C	4.076503	2.652929	2.750215
C	4.194827	2.196588	1.433799
C	3.210072	2.442101	0.476649
C	0.739007	4.483129	2.585597
C	5.170412	2.401156	3.755672
C	3.387563	1.964489	-0.938218

H	-2.853009	4.887161	-3.164602
H	-1.688228	4.344017	-4.355639
H	-1.437523	5.893378	-3.529428
H	0.169744	5.901571	-2.145152
H	1.985733	6.431194	-1.162939
H	3.070963	5.144724	-0.596455
H	2.005759	5.950583	0.541527
H	-5.689233	1.756493	-2.893196
H	-2.650173	0.408215	-5.593698
H	-3.675255	4.091817	-1.116467
H	-3.839876	2.581683	-0.237774
H	-5.212685	3.213265	-1.149017
H	-6.340054	0.694824	-4.975899
H	-5.136577	0.398430	-6.239437
H	-5.374414	-0.782898	-4.953107
H	-0.406983	0.959751	-5.289464
H	-0.114066	2.530619	-4.528436
H	0.121328	1.056761	-3.604482
H	2.816717	3.740399	4.107888
H	5.082799	1.639935	1.138698
H	-0.142642	4.271678	1.978410
H	0.485791	4.298169	3.632083
H	0.953929	5.555235	2.486258
H	5.537266	1.370335	3.701182
H	4.825349	2.586108	4.777388
H	6.032496	3.056404	3.579400
H	4.356330	1.476806	-1.069553
H	3.320506	2.789267	-1.656283
H	2.615535	1.238406	-1.224816
C	1.578351	-3.018704	2.006810
C	1.671044	-0.792534	2.528321
C	2.261638	-3.373502	3.153936
H	1.247314	-3.785811	1.309654
C	2.357999	-1.039193	3.701908

H	1.418224	0.227995	2.256352
H	2.455369	-4.422297	3.341356
H	2.627245	-0.197390	4.326267
N	1.272057	-1.751372	1.676665
C	2.678571	-2.368677	4.059796
N	3.348231	-2.666002	5.210183
C	3.683421	-1.609557	6.146457
H	2.787946	-1.101458	6.527757
H	4.216457	-2.040388	6.994307
H	4.336603	-0.859147	5.685356
C	3.605680	-4.048342	5.564613
H	4.173372	-4.078383	6.494893
H	2.676779	-4.615245	5.713783
H	4.200032	-4.554146	4.794131
C	-1.904028	1.502304	1.410064
H	-2.828028	1.003059	1.114226
H	-0.865874	0.514075	0.568671
C	-1.628430	1.245494	2.831469
F	-1.571781	-0.076315	3.125361
F	-2.546562	1.761131	3.719131
F	-0.436983	1.767815	3.241122
H	-1.991734	2.585033	1.253848

TS7.log

Mg	-0.064510	-1.242445	0.999117
N	1.106473	-2.561794	-0.224006
N	-1.526473	-2.780768	1.267247
C	1.877783	-4.742278	-1.070723
C	0.930393	-3.876221	-0.261045
C	-0.129296	-4.562220	0.371551
C	-1.311380	-4.055558	0.953952
C	-2.412304	-5.079278	1.165358
C	2.083017	-1.999995	-1.098087
C	3.405561	-1.775733	-0.672673
C	4.323388	-1.223140	-1.572366
C	3.970221	-0.870726	-2.874351
C	2.649025	-1.092726	-3.271076
C	1.701502	-1.650202	-2.411291
C	3.845209	-2.121744	0.724256
C	4.970799	-0.256692	-3.818904
C	0.289298	-1.876741	-2.875634
C	-2.833141	-2.436245	1.731029
C	-3.164307	-2.531549	3.097027
C	-4.456655	-2.193533	3.507520
C	-5.429225	-1.753351	2.609326
C	-5.074812	-1.659460	1.262761
C	-3.796702	-1.988498	0.806244
C	-2.158654	-3.013238	4.108957
C	-6.804676	-1.360277	3.081201
C	-3.457706	-1.877094	-0.654565
H	2.922418	-4.527631	-0.827128
H	1.762207	-4.540507	-2.141165
H	1.685613	-5.803322	-0.900669
H	-0.115363	-5.637825	0.237988
H	-2.049686	-6.091187	0.976633
H	-3.242948	-4.880922	0.478771
H	-2.830886	-5.035370	2.174002
H	5.349174	-1.069319	-1.240228
H	2.345461	-0.832069	-4.283868
H	3.514928	-3.120981	1.024678
H	3.424960	-1.420350	1.452683
H	4.935224	-2.081144	0.809133
H	5.996826	-0.417943	-3.474667
H	4.884396	-0.679217	-4.825685
H	4.822338	0.826385	-3.911368
H	0.165001	-1.565955	-3.916533
H	-0.007330	-2.928577	-2.796774

H	-0.416700	-1.305710	-2.260163
H	-4.708143	-2.279438	4.563768
H	-5.814632	-1.320568	0.539479
H	-2.567537	-2.953056	5.121941
H	-1.860847	-4.054151	3.933918
H	-1.239009	-2.422309	4.070414
H	-7.563624	-1.562986	2.318822
H	-6.853040	-0.288540	3.310747
H	-7.087443	-1.900847	3.990053
H	-4.326052	-1.555256	-1.234289
H	-3.099857	-2.827570	-1.065819
H	-2.659670	-1.142514	-0.819638
Mg	-0.278864	1.721573	0.394452
N	-1.369474	2.941552	-0.930967
N	0.951757	3.363573	0.934975
C	-2.018747	5.003092	-2.108149
C	-1.176253	4.246416	-1.099789
C	-0.200360	5.008666	-0.426566
C	0.814496	4.599472	0.456481
C	1.831174	5.662879	0.825507
C	-2.310222	2.293479	-1.787693
C	-3.660799	2.175433	-1.410772
C	-4.549556	1.531569	-2.277103
C	-4.138671	0.989244	-3.495146
C	-2.790473	1.108985	-3.840447
C	-1.867869	1.749783	-3.011239
C	-4.148436	2.733101	-0.101277
C	-5.109991	0.277792	-4.401117
C	-0.425347	1.868894	-3.422847
C	2.070480	3.090342	1.772165
C	2.001162	3.313228	3.161213
C	3.112796	3.010740	3.956836
C	4.289201	2.491099	3.417607
C	4.331414	2.269048	2.037957
C	3.248717	2.556174	1.205771
C	0.762814	3.896831	3.785439
C	5.481130	2.178273	4.284825
C	3.337974	2.312001	-0.275020
H	-3.086855	4.870185	-1.912085
H	-1.841958	4.625337	-3.120762
H	-1.789647	6.069921	-2.092933
H	-0.179874	6.059276	-0.691107
H	1.556312	6.633156	0.408724
H	2.820256	5.391122	0.440245
H	1.939574	5.765988	1.908695
H	-5.596380	1.456207	-1.987139
H	-2.444303	0.697935	-4.787513
H	-3.860732	3.781379	0.033072
H	-3.731442	2.175185	0.742701
H	-5.238006	2.668721	-0.033125
H	-5.037992	-0.811162	-4.287959
H	-6.143594	0.561155	-4.180855
H	-4.916160	0.504724	-5.454682
H	-0.263926	1.424065	-4.408633
H	-0.094275	2.912718	-3.463329
H	0.234450	1.358802	-2.709809
H	3.056062	3.203355	5.027660
H	5.238578	1.865751	1.590962
H	-0.135800	3.421364	3.385340
H	0.774410	3.775935	4.872621
H	0.663062	4.968982	3.576013
H	5.713552	1.106389	4.276375
H	5.309644	2.476788	5.323528
H	6.378953	2.700476	3.935117
H	4.335652	1.965751	-0.555679
H	3.110130	3.215676	-0.850639
H	2.620026	1.544405	-0.592131
C	1.330890	-2.805332	3.274498

C	1.581402	-0.556931	3.610126
C	2.089502	-3.113725	4.388185
H	0.913644	-3.600860	2.660521
C	2.348772	-0.754829	4.743757
H	1.376665	0.451215	3.257491
H	2.254536	-4.155426	4.633866
H	2.731409	0.113156	5.265181
N	1.061855	-1.550983	2.873689
C	2.628912	-2.071320	5.178076
N	3.377703	-2.322080	6.290793
C	3.899994	-1.221775	7.077356
H	3.094929	-0.586200	7.467896
H	4.454159	-1.621323	7.926967
H	4.583292	-0.592657	6.492899
C	3.650625	-3.688986	6.690485
H	4.266194	-3.681972	7.590246
H	2.727606	-4.238204	6.916982
H	4.196894	-4.237142	5.912273
C	-2.306799	1.547687	3.346223
C	-1.840782	0.936143	4.606880
F	-1.160320	0.837695	1.974815
F	-3.531372	1.163074	2.966071
H	-1.878521	-0.153778	4.539195
H	-2.474940	1.242133	5.456149
H	-0.814681	1.244985	4.815199
H	-2.146502	2.602746	3.141992

TS8.log

Mg	0.157345	-1.357447	-0.169125
N	1.188987	-2.578340	-1.570694
N	-1.347011	-2.831828	0.040555
C	1.934119	-4.744315	-2.475493
C	1.007284	-3.897251	-1.623254
C	-0.022685	-4.597050	-0.963782
C	-1.178282	-4.096074	-0.316974
C	-2.288424	-5.101414	-0.082009
C	2.095188	-1.996020	-2.503950
C	3.420405	-1.687915	-2.140597
C	4.262800	-1.091138	-3.084025
C	3.834295	-0.782147	-4.374943
C	2.517313	-1.099838	-4.714671
C	1.641314	-1.698962	-3.807323
C	3.942001	-2.007699	-0.766087
C	4.751890	-0.110985	-5.363434
C	0.231961	-2.030982	-4.217419
C	-2.615765	-2.432764	0.562202
C	-2.887295	-2.508748	1.943021
C	-4.124981	-2.063316	2.411626
C	-5.098452	-1.535995	1.560645
C	-4.806083	-1.473896	0.197812
C	-3.582756	-1.911542	-0.317921
C	-1.868703	-3.046036	2.911315
C	-6.408219	-1.028483	2.104841
C	-3.303504	-1.819928	-1.792790
H	2.983468	-4.512229	-2.269670
H	1.776395	-4.546206	-3.540933
H	1.766713	-5.808536	-2.299774
H	-0.015209	-5.670196	-1.117182
H	-1.960821	-6.115471	-0.317862
H	-3.148971	-4.863290	-0.717089
H	-2.649528	-5.074499	0.949610
H	5.289979	-0.869397	-2.798226
H	2.158861	-0.879843	-5.719098
H	3.750439	-3.049537	-0.488578
H	3.458474	-1.392219	-0.000633
H	5.020306	-1.832787	-0.709640
H	4.570495	-0.464601	-6.383502

H	4.606936	0.976661	-5.368211
H	5.803300	-0.297692	-5.124547
H	0.043739	-1.722161	-5.249182
H	0.022554	-3.103992	-4.144678
H	-0.497816	-1.531840	-3.571033
H	-4.327539	-2.121904	3.479496
H	-5.548640	-1.073520	-0.490267
H	-2.310351	-3.173114	3.903530
H	-1.461347	-4.011269	2.592764
H	-1.026458	-2.355328	3.008196
H	-7.158013	-0.927469	1.314431
H	-6.286766	-0.043979	2.572730
H	-6.812809	-1.700261	2.869325
H	-4.172078	-1.435119	-2.331963
H	-3.031494	-2.790645	-2.221887
H	-2.465382	-1.141717	-1.994217
Mg	-0.310472	1.810384	-0.572064
N	-1.310699	2.889922	-2.035475
N	0.970914	3.378739	-0.100589
C	-1.765091	4.852923	-3.446078
C	-0.988475	4.142653	-2.355510
C	0.051982	4.887284	-1.765664
C	0.969015	4.537525	-0.757864
C	2.029527	5.572689	-0.441877
C	-2.314370	2.243175	-2.821798
C	-3.668595	2.279293	-2.440821
C	-4.610577	1.630273	-3.246238
C	-4.252842	0.943318	-4.406318
C	-2.901471	0.920832	-4.760387
C	-1.925811	1.558444	-3.992323
C	-4.118142	3.013273	-1.205296
C	-5.284991	0.235338	-5.245008
C	-0.483657	1.540867	-4.421877
C	1.933721	3.183861	0.931564
C	1.697497	3.665038	2.235738
C	2.642835	3.403050	3.234129
C	3.819155	2.698987	2.977549
C	4.033020	2.237280	1.675546
C	3.114716	2.464073	0.648975
C	0.480423	4.491171	2.562529
C	4.847839	2.477283	4.056261
C	3.392086	1.970954	-0.744588
H	-2.832960	4.882470	-3.208177
H	-1.676408	4.325276	-4.400997
H	-1.409901	5.875619	-3.580178
H	0.184017	5.882318	-2.173136
H	1.950422	6.433205	-1.107715
H	3.030240	5.140965	-0.546831
H	1.949919	5.927181	0.589756
H	-5.659314	1.674115	-2.957043
H	-2.596176	0.400982	-5.666975
H	-3.637477	3.991657	-1.109882
H	-3.877096	2.453270	-0.295613
H	-5.201017	3.165325	-1.219139
H	-5.082635	0.353929	-6.314507
H	-5.297572	-0.841837	-5.037352
H	-6.290963	0.617541	-5.048280
H	-0.361283	0.980131	-5.351852
H	-0.091628	2.551298	-4.583720
H	0.159243	1.077061	-3.664226
H	2.453138	3.774591	4.240031
H	4.945230	1.688559	1.447337
H	-0.378946	4.212854	1.948460
H	0.205466	4.389878	3.616398
H	0.663295	5.558452	2.382712
H	5.322289	1.494673	3.963651
H	4.405410	2.550601	5.054481
H	5.648517	3.225437	4.001343

H	4.369088	1.484861	-0.801883
H	3.373870	2.787071	-1.475173
H	2.640121	1.242155	-1.074194
C	1.636342	-3.070841	1.929925
C	1.808090	-0.856427	2.473870
C	2.323679	-3.460649	3.063264
H	1.271494	-3.818971	1.229100
C	2.504136	-1.137485	3.634561
H	1.585690	0.174033	2.212565
H	2.485438	-4.517259	3.236476
H	2.809822	-0.312984	4.265242
N	1.366149	-1.790984	1.618050
C	2.785087	-2.480653	3.974131
N	3.459632	-2.813096	5.112536
C	3.833195	-1.780545	6.060292
H	2.956242	-1.254342	6.460147
H	4.364733	-2.237932	6.895095
H	4.501237	-1.041064	5.602780
C	3.668339	-4.207510	5.451783
H	4.248265	-4.267568	6.373052
H	2.720587	-4.739830	5.609847
H	4.231651	-4.728447	4.668355
C	-1.827793	1.505570	1.398795
H	-2.720724	0.901911	1.215611
H	-0.802814	0.497581	0.559289
C	-1.415766	1.432945	2.811334
F	-1.071389	0.143384	3.173855
F	-2.427351	1.780971	3.719334
H	-2.031837	2.553838	1.135836
H	-0.564544	2.069250	3.071880

TS9.log

Mg	0.006348	-1.364841	-0.199057
N	1.105377	-2.519254	-1.587932
N	-1.431528	-2.896027	0.027221
C	1.969596	-4.658980	-2.452058
C	1.005684	-3.848129	-1.606248
C	0.028342	-4.593375	-0.918027
C	-1.159587	-4.156654	-0.285251
C	-2.184158	-5.240458	-0.010223
C	1.992411	-1.918427	-2.529535
C	3.313184	-1.585861	-2.173343
C	4.148910	-1.006528	-3.133164
C	3.714353	-0.731967	-4.429960
C	2.398361	-1.065090	-4.758138
C	1.530141	-1.652493	-3.835665
C	3.833851	-1.858404	-0.788116
C	4.624343	-0.077576	-5.436458
C	0.123529	-2.009276	-4.234347
C	-2.741022	-2.610597	0.527332
C	-3.012203	-2.669575	1.907235
C	-4.306669	-2.393255	2.354285
C	-5.335808	-2.044916	1.479031
C	-5.037836	-1.982317	0.117501
C	-3.759986	-2.253005	-0.375387
C	-1.934946	-3.021018	2.897757
C	-6.713188	-1.712152	1.989606
C	-3.479547	-2.153385	-1.850223
H	3.007354	-4.367227	-2.265629
H	1.787566	-4.488700	-3.518533
H	1.861512	-5.727051	-2.255237
H	0.108391	-5.667431	-1.039770
H	-1.771553	-6.231620	-0.206174
H	-3.060344	-5.100700	-0.652881
H	-2.549287	-5.203113	1.019641
H	5.174579	-0.768612	-2.855396
H	2.035152	-0.868986	-5.765672

H	3.656237	-2.893950	-0.479488
H	3.339940	-1.224191	-0.044268
H	4.909232	-1.666507	-0.732795
H	4.478665	1.009682	-5.457624
H	5.677592	-0.260032	-5.202404
H	4.435021	-0.448083	-6.448985
H	-0.067510	-1.734204	-5.275090
H	-0.074875	-3.081569	-4.127473
H	-0.611741	-1.495570	-3.605672
H	-4.513582	-2.452202	3.421922
H	-5.821144	-1.709082	-0.587437
H	-2.348004	-3.099748	3.907679
H	-1.443535	-3.970911	2.659910
H	-1.150947	-2.257070	2.909223
H	-7.487965	-1.997276	1.270737
H	-6.817380	-0.634933	2.169166
H	-6.925746	-2.222032	2.934530
H	-4.398013	-1.955063	-2.408335
H	-3.025774	-3.067267	-2.248546
H	-2.783559	-1.333466	-2.062228
Mg	-0.302222	1.939136	-0.465551
N	-1.147586	3.042150	-2.014776
N	1.183218	3.372178	-0.118094
C	-1.506056	5.031490	-3.413382
C	-0.768265	4.279297	-2.323795
C	0.301150	4.970197	-1.719359
C	1.218957	4.549739	-0.739323
C	2.329137	5.529039	-0.412182
C	-2.187594	2.443971	-2.788654
C	-3.529429	2.527824	-2.370324
C	-4.512135	1.896624	-3.138490
C	-4.207600	1.186886	-4.300898
C	-2.868154	1.114034	-4.689668
C	-1.851608	1.730547	-3.956447
C	-3.908250	3.287188	-1.127152
C	-5.291979	0.548360	-5.129777
C	-0.419987	1.646174	-4.412152
C	2.197045	3.080973	0.838519
C	2.035687	3.443919	2.190792
C	3.043650	3.108299	3.102898
C	4.200627	2.430124	2.718382
C	4.331734	2.072899	1.373606
C	3.353807	2.385012	0.427920
C	0.823498	4.209033	2.651442
C	5.291668	2.115678	3.709239
C	3.535089	2.003564	-1.015120
H	-2.575704	5.091997	-3.190043
H	-1.419999	4.514307	-4.374403
H	-1.115416	6.043708	-3.528804
H	0.469750	5.969955	-2.101424
H	2.217053	6.455908	-0.976703
H	3.306953	5.096173	-0.649113
H	2.347940	5.771161	0.654549
H	-5.548959	1.965764	-2.813716
H	-2.603464	0.565515	-5.592309
H	-3.513153	4.308858	-1.136947
H	-3.515614	2.803474	-0.228104
H	-4.995742	3.344992	-1.027706
H	-5.631778	1.220521	-5.928054
H	-4.942020	-0.371157	-5.609547
H	-6.167049	0.299571	-4.521757
H	-0.335519	1.049561	-5.324069
H	0.003635	2.635894	-4.617713
H	0.221649	1.189621	-3.649940
H	2.921560	3.404822	4.143874
H	5.225153	1.543608	1.046077
H	-0.103641	3.722640	2.338148
H	0.817821	4.305851	3.740982

H	0.801779	5.221694	2.231092	C	3.510142	-1.248875	6.116674
H	5.605342	1.067571	3.644469	H	2.580202	-0.782877	6.468220
H	4.968635	2.311566	4.736331	H	4.050317	-1.623603	6.986342
H	6.184358	2.726463	3.527241	H	4.127435	-0.475299	5.644676
H	4.487017	1.489724	-1.168901	C	3.611541	-3.700474	5.593393
H	3.510184	2.878754	-1.673738	H	4.161575	-3.668655	6.534103
H	2.736691	1.336061	-1.360055	H	2.727796	-4.336469	5.737337
C	1.564275	-2.902213	1.988871	H	4.258445	-4.171569	4.843337
C	1.504010	-0.661473	2.448747	C	-1.868209	0.855570	1.730933
C	2.250297	-3.180747	3.155605	H	-0.790361	0.272366	0.540320
H	1.293713	-3.706893	1.308146	C	-2.034980	1.049240	3.212738
C	2.183160	-0.830626	3.640659	F	-1.433005	2.234315	1.237735
H	1.198022	0.331996	2.130837	F	-3.112664	0.767358	1.161767
H	2.512610	-4.208534	3.373231	H	-2.720741	1.875018	3.457651
H	2.402024	0.044954	4.237797	H	-1.065172	1.238000	3.680483
N	1.182148	-1.667614	1.620148	H	-2.443595	0.123906	3.629257
C	2.583528	-2.127238	4.039775				
N	3.251426	-2.349625	5.207969				

5. ^{19}F NMR Spectroscopic Data

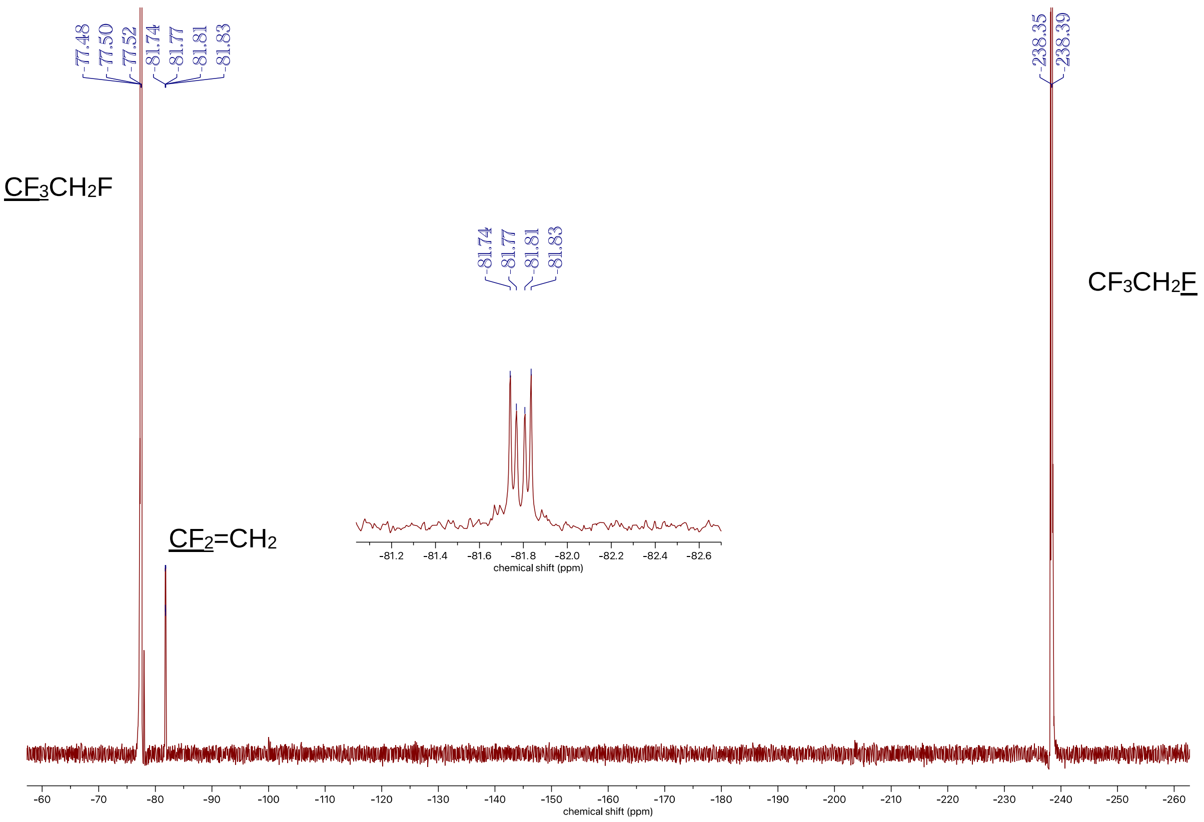

Figure S6: ^{19}F NMR Spectrum of the volatiles from the reaction mixture showing unreacted starting material and 1,1-difluoroethene.

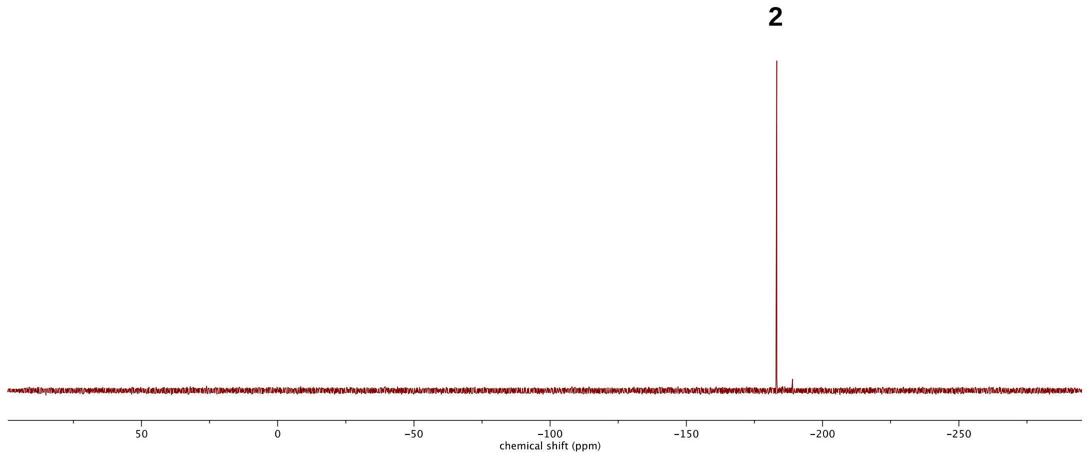

Figure S7: ^{19}F NMR Spectrum of the reaction of **1** + DMAP with HFC-134a showing **2** following removal of the volatiles.

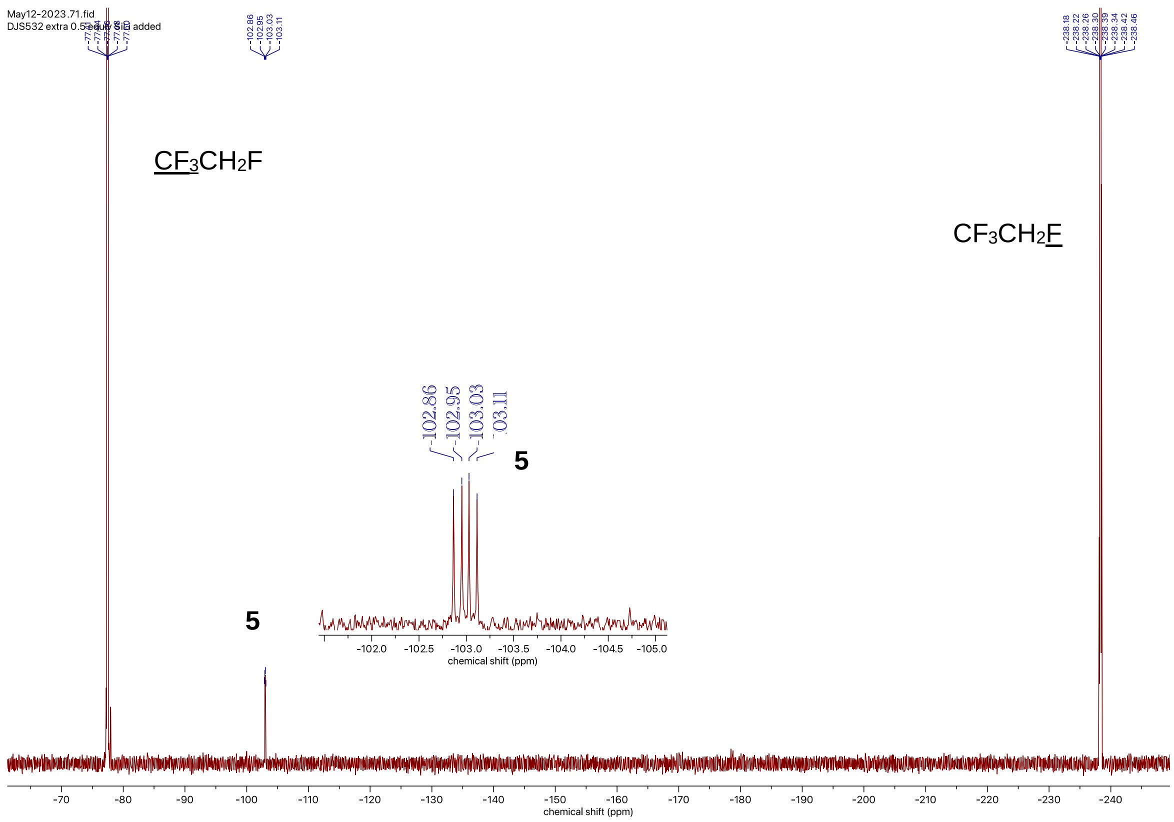

Figure S8: ^{19}F NMR Spectrum of the reaction of the two-step reaction of **1** + DMAP followed by addition of **4** showing, **5** and unreacted HFC-134a.

6. References

[1] J. Hicks, M. Juckel, A. Paparo, D. Dange, C. Jones, *Organometallics* **2018**, *37*, 4810–4813.

[2] D. J. Sheldon, J. M. Parr, M. R. Crimmin, *J. Am. Chem. Soc.* **2023**, *145*, 10486–10490.

[3] N. O. Andrella, N. Xu, B. M. Gabidullin, C. Ehm, R. T. Baker, *J. Am. Chem. Soc.* **2019**, *141*, 11506–11521.

[4] C. Bakewell, B. J. Ward, A. J. P. White, M. R. Crimmin, *Chem. Sci.* **2018**, *9*, 2348–2356.

[5] M. Ohashi, H. Saijo, M. Shibata, S. Ogoshi, *European J. Org. Chem.* **2013**, *2013*, 443–447.

[6] H. Sakaguchi, M. Ohashi, S. Ogoshi, *Angew. Chem., Int. Ed.* **2018**, *57*, 328–332.

[7] M. Ohashi, H. Saijo, M. Shibata, S. Ogoshi, *European J. Org. Chem.* **2013**, *2013*, 443–447.

[8] T. Hanamoto, Y. Kiguchi, K. Shindo, M. Matsuoka, M. Kondo, *Chem. Commun.* **1999**, 151–152.

[9] M. J. Frisch, G. W. Trucks, H. B. Schlegel, G. E. Scuseria, M. A. Robb, J. R. Cheeseman, G. Scalmani, V. Barone, B. Mennucci, G. A. Petersson, Gaussian 09; Revision D.01, Gaussian 09; Revision D.01, Gaussian Inc., **2009**.

[10] J. P. Perdew, K. Burke, Y. Wang, *Phys. Rev. B* **1996**, *54*, 16533–16539.

[11] A. D. Becke, *J. Chem. Phys.* **1993**, *98*, 5648–5652.

[12] J. P. Perdew, K. Burke, M. Ernzerhof, *Phys. Rev. Lett.* **1996**, *77*, 3865–3868.

[13] J. P. Perdew, J. A. Chevary, S. H. Vosko, K. A. Jackson, M. R. Pederson, D. J. Singh, C. Fiolhais, *Phys. Rev. B* **1993**, *48*, 4978.

[14] J. P. Perdew, J. A. Chevary, S. H. Vosko, K. A. Jackson, M. R. Pederson, D. J. Singh, C. Fiolhais, *Phys. Rev. B* **1992**, *46*, 6671–6687.

[15] Y. Zhao, D. G. Truhlar, *Theor. Chem. Acc.* **2008**, *120*, 215–241.

[16] A. Lledós, *Eur. J. Inorg. Chem.* **2021**, *2021*, 2547–2555.

[17] F. Weigend, R. Ahlrichs, *Phys. Chem. Chem. Phys.* **2005**, *7*, 3297–3305.

[18] J. Tomasi, B. Mennucci, R. Cammi, *Chem. Rev.* **2005**, *105*, 2999–3094.

[19] S. Grimme, J. Antony, S. Ehrlich, H. Krieg, *J. Chem. Phys.* **2010**, *132*, 154104.

[20] S. Grimme, S. Ehrlich, L. Goerigk, *J. Comput. Chem.* **2011**, *32*, 1456–1465.

[21] K. Fukui, *Acc. Chem. Res.* **1981**, *14*, 363–368.

[22] C. E. Dykstra, *Theory and Applications of Computational Chemistry : The First Forty Years*, Elsevier, **2005**.

[23] R. Dennington, T. Keith, J. Milliam, GaussView 5.0, GaussView 5.0, Semichem Inc., Shawnee Mission, KS, **2009**.

[24] E. D. Glendening, J. K. Badenhoop, A. E. Reed, J. E. Carpenter, J. A. Bohmann, C. M. Morales, C. R. Landis, F. Weinhold, NBO 6.0, NBO 6.0, Theoretical Chemistry Institute, University of Wisconsin, Madison, **2013**.

[25] J.-D. Chai, M. Head-Gordon, *J. Chem. Phys.* **2008**, *128*, 84106.

[26] J.-D. Chai, M. Head-Gordon, *Phys. Chem. Chem. Phys.* **2008**, *10*, 6615–6620.

[27] G. Coates, B. J. Ward, C. Bakewell, A. J. P. White, M. R. Crimmin, *Chem. - A Eur. J.* **2018**, *24*, 16282–16286.
